# Millimeter‐Scale Soft Continuum Robots for Large‐Angle and High‐Precision Manipulation by Hybrid Actuation

**DOI:** 10.1002/aisy.202000189

**Published:** 2020-11-19

**Authors:** Tieshan Zhang, Liu Yang, Xiong Yang, Rong Tan, Haojian Lu, Yajing Shen

**Affiliations:** ^1^ Department of Biomedical Engineering City University of Hong Kong Tat Chee Avenue Kowloon Hong Kong China; ^2^ The State Key Laboratory of Industrial Control and Technology Zhejiang University Hangzhou 310027 China; ^3^ Shenzhen Research Institute of City University of Hong Kong Shenzhen 518057 China

**Keywords:** magnetic actuations, micromanipulations, millimeter-scale soft continuum robots, tendon driven robots

## Abstract

Developing small‐scale soft continuum robots with large‐angle steering capacity and high‐precision manipulation offers broad opportunities in various biomedical settings. However, existing continuum robots reach the bottleneck in actuation on account of the contradiction among small size, compliance actuation, large tender range, high precision, and small dynamic error. Herein, a 3D‐printed millimeter‐scale soft continuum robot with an ultrathin hollow skeleton wall (300 μm) and a large inner‐to‐outer ratio (0.8) is reported. After coating a thin ferromagnetic elastomer layer (≈100–150 μm), the proposed soft continuum robot equipped with hybrid actuation (tendon‐ and magnetic‐driven mode) achieves large‐angle (up to 100°) steering and high‐precision (low to 2 μm for static positioning) micromanipulation simultaneously. Specifically, the robot implements an ultralow dynamic tracking error of ≈10 μm, which is ≈30‐fold improved than the state of art. Combined with a microneedle/knife or nasopharyngeal swab, the robot reveals the potential for versatile biomedical applications, such as drug injection on the target tissue, diseased tissue ablation, and COVID‐19 nasopharyngeal sampling. The proposed millimeter‐scale soft continuum robot presents remarkable advances in large‐range and high‐precise actuation, which provides a new method for miniature continuum robot design and finds broad applications in biomedical engineering.

## Graphical abstract

Developing small‐scale soft continuum robots offers broad opportunities in various biomedical settings. Herein, a 3D‐printed millimeter‐scale soft continuum robot equipped with hybrid actuation (tendon and magnetic driven) that can achieve large‐angle (≈100°) steering and high‐precision (≈2/10 μm for static/dynamic positioning, respectively) micromanipulation simultaneously is proposed, which reveals versatile potentials, such as microinjection/ablation and nasopharyngeal sampling.

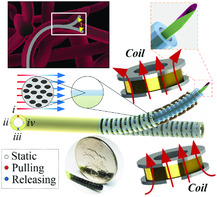

## 1 Introduction

**Figure 1:**
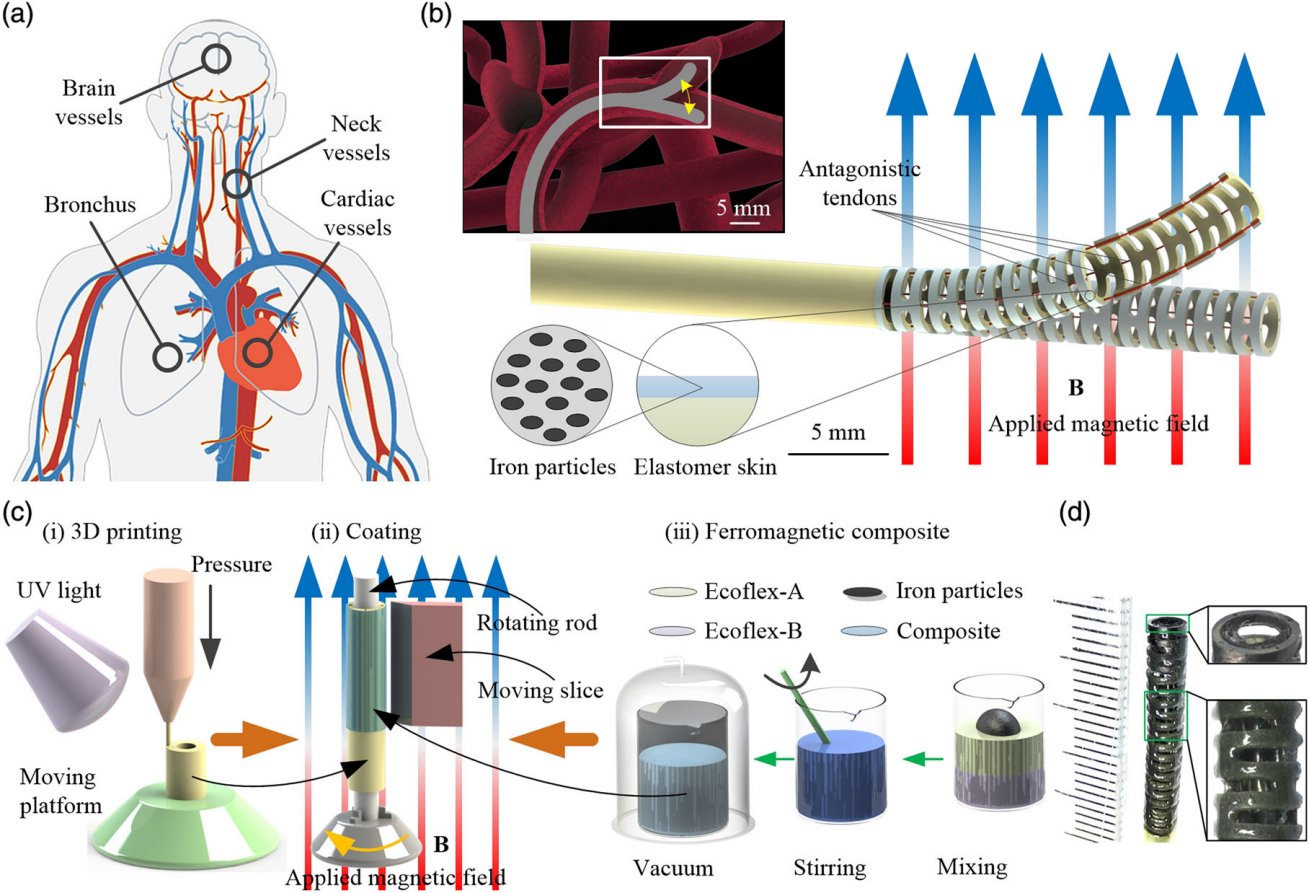
Illustration of small‐scale soft continuum robot design with hybrid magnetic‐ and tendon‐driven mode. a) Pathologic areas across the human body hard to reach, where small‐scale soft steering robots can present their superiority. b) Illustration of steering ability of a small‐scale soft continuum robot catheter passing through a complex constrained vascular environment. The enlarged schematic of the magnetic‐ and tendon‐driven soft continuum robot with an elastomer skin grown onto the hollow skeleton. The elastomer skin consists of silicone rubber embedded with evenly rearranged iron particles. c) The schematic illustration of the fabrication process for the soft continuum robot. (i) The micro‐3D printing process by which the detailed structure of the catheter skeleton is fabricated. (ii) The illustration of the coating process. The ferromagnetic elastomer skin is brushed onto the outer surface of the continuum robot and rearranged under external applied magnetic field **B**. (iii) The procedures of making ferromagnetic elastomer skin which consists of silicone rubber (Ecoflex) and microiron particles with a prescribed mass fraction. d) The optical image of the fabricated soft continuum robot. The spacing here represents 1 mm.

Small‐scale continuum robots with soft body own great application potentials^[^ ^1^, ^2^, ^3^ ^]^ on various pathological areas inside the human body (**Figure** **1**a) due to their advances in steering and navigation capacities within constrained environments. To conduct effective operations in the abovementioned areas, the contour of the robot should maintain a millimeter scale whereas a large inner lumen shall be granted for carrying surgical tools. Moreover, to achieve friendly contact and easily pass through within the complex constrained environment (e.g., blood vessels) (Figure 1b), inherent compliance and a larger tender range are essentially needed for the robot when searching for pathological areas. In addition, the merits of higher precision and smaller dynamic error are also necessarily required for the robot to implement a safer and more effective target therapy in vivo.

To control the continuum robots to follow the desired trajectories, multiple actuation mechanisms have been developed. Tendon‐driven robots,^[^ ^4^, ^5^, ^6^ ^]^ with a relatively rigid main body actuated via pairs of cables, have appealed to favor of many scholars. Based on the multisectional tubular structure, tendon‐driven robots can be easily established via analytical kinematic expressions and utilized for delivering smaller surgery tools, which benefits posture prediction and multitasking operation in endoscopic surgical procedures. This type of actuation can usually reach a steering angle of ≈100° and achieve the positioning precision of around 2.0 mm. However, traditional tendon‐driven continuum robots have the difficulty of scaling down to a small scale while keeping a large inner lumen through conventional manufacturing technology. For reducing the components and rigidity of the end‐effector and maintaining a large workspace simultaneously, the fluid‐driven mechanism^[^ ^7^, ^8^, ^9^ ^]^ has been proposed as a substitute. Although the soft body grants the fluidic actuation robots with the merits of friendly contact and excellent bending angle (>150), the complexity of the control system keeps increasing^[^ ^10^ ^]^ due to the nonlinear properties. Therefore, precise positioning is hard to achieve. In addition, the fluid‐driven robots remain in the contour, even larger than the tendon‐driven ones.

For achieving a small‐scale contour, numerous advanced materials have been proposed and demonstrate unique superiority.^[^ ^11^, ^12^ ^]^ Specifically, soft continuum robots embedded with tiny magnets^[^ ^13^, ^14^, ^15^ ^]^ or fabricated via ferromagnetic composite^[^ ^16^ ^]^ demonstrate precise steering capability (bending angle range from 90° to 180° and positioning precision up to 0.3 mm^[^ ^13^ ^]^) under an external controllable magnetic field. Usually, the magnetic actuated soft continuum robots’ diameters are smaller than 3.0 mm, which ensure their capabilities of endoscopic imaging inside the bronchus or target laser ablation inside a cerebrovascular phantom network. However, magnetic actuated soft continuum robots are hard to keep steady when they suffer from an external force, e.g., the resistance from the channel wall. Morevoer, the proposed robot body without inner lumen limits the robots’ function integration. In addition, the tiny rigid magnet tip suffers the risk of breaking off in vivo during manipulation. To achieve safer and direct actuation, shape‐memory materials^[^ ^17^, ^18^ ^]^ have been discovered and widely applied as actuators for continuum soft robots to accomplish specific tasks, i.e., cardiovascular inspection and nasopharyngeal drug delivery. The self‐deformation material can achieve actuation with the whole body while maintaining a small scale in the meantime. However, it is hard to realize quick response and precise positioning simultaneously due to the inherent hysteresis phenomenon. Although shape‐memory material‐actuated soft continuum robots have potentials to be developed down to the millimeter scale, the large steering angle is still remains a challenge.

To achieve precise posture prediction and variable stiffness against different environments, scholars developed some manipulators^[^ ^19^, ^20^ ^]^ combining pneumatic and tendons. This type of hybrid‐actuation robot demonstrates the features of friendly gripping through mimicking the octopus’ muscles and performs the bending angle larger than 90 °. However, there remain shortcomings, such as the relative large‐scale size, low static and dynamic precision, and the complex control strategy. To improve compliance and decrease the complexity of the actuation system, a shape memory polymer‐based fluid actuated arm^[^ ^21^ ^]^ was proposed to be applied in minimally invasive surgery (MIS). Although the actuation is reported to be able to achieve helical bending (≈140°) and variable stiffness, the properties of centimeter‐scale contour and thermal hysteresis exist.

Considering the circumstances that most of the existing continuum robots find it hard to achieve a comprehensive performance of large‐angle steering and high‐precision manipulation while maintaining small‐scale size, here, we propose a millimeter‐scale soft continuum robot with the hybrid‐actuation mode to tackle the problem (Figure 1b). Tendon‐driven mechanisms could achieve relatively large‐angle steering control and resist higher external forces for searching pathological areas in vivo. A precisely controlled external magnetic field is capable of leading high‐precision manipulation after arrival. With the help of micro‐3D printing technology, the skeleton with an outer/inner diameter of 3.0/2.4 mm could be fabricated precisely (Figure 1c‐i). Four through‐holes (diameter of 150 μm) can also be constructed within the ultrathin wall (300 μm). To integrate magnetic actuation and increase compliance, we grow an elastomer skin layer (thickness of ≈100–150 μm) embedded with iron particles onto the surface of the robot, as shown in Figure 1c‐ii. With proper pull–release strategies of tendons, the robot can achieve a bending angle up to 100° and reach a large workspace. Under the actuation of the magnetic field, the precision of static positioning down to 2 μm and a low dynamic tracking error at ≈10 μm are finally achieved. With a combination of both tendon driven and magnetic actuation, the proposed soft continuum robot demonstrated the excellent capability of steering within the vessel‐mimicked‐branched tunnel and precise tracking. We further demonstrate the versatile micromanipulation capabilities through equipping the microtools to present more potential applications with relevance to clinical surgery, e.g., microinjection/ablation and nasopharyngeal sampling.

## 2 Results and Discussion

### 2.1 Hybrid‐Actuation Millimeter‐Scale Soft Continuum Robot Design

To obtain a large bending angle and predict the posture accurately, a tendon‐driven mechanism is used as the actuation for the robot during the period of navigating to the pathological area. For the sake of achieving the steering capacity along the plane perpendicular to its central axis, two pairs of antagonistic tendons are applied. Four through‐holes are constructed within the wall to implement the tendons (a stainless steel wire with a diameter of 100 μm), placed at 90° between each other. To adapt to the circumstances within vessels in vivo, the outer diameter of the skeleton is designed as 3.0 mm, and a hollow structure (Figure S1a, Supporting Information) along the bending directions is designed to increase the compliance of the robot. Furthermore, the designed large inner lumen promotes the robot to explore versatile capacities via encapsulating varied surgical tools.

Here, the micro‐3D printing system (nanoArch P140, BMF Material Technology Inc.) with a printing precision of 10 μm is used to fabricate such a delicate robot skeleton (Figure 1c‐i). For obtaining enough strength and elasticity of the structure, a type of ductile photosensitive resin (HD, BMF Material Technology Inc.) with an elastic modulus of 3.6 GPa is utilized as the printing material. As shown in Table S1, Supporting Information, the material possesses the breaking elongation rate of 24.3%, leading to a low risk of breaking off for large deformation. The skeleton is printed with an outer/inner diameter of D/d=3.0/2.4 mm, hollow section of a=500 μm, and through‐holes of $d_{h}=150$ μm (Figure S1b, Supporting Information). As a result, an ultrathin wall of 300 μm is obtained, which is the thinnest wall among all reported continuum tendon‐driven robots.^[^ ^22^ ^]^ Moreover, the large inner‐to‐outer ratio, i.e., 0.8, grants the robot high superiority in encapsulating versatile small surgical tools while maintaining a tiny size (Figure 1b). Meanwhile, the hollow skeleton allows the robot to easily achieve a large bending motion while keeping enough stiffness.

To achieve high‐precise manipulation and maintain friendly interaction with the tissue, we decorate a thin layer of ferromagnetic composite elastomer skin (≈100–150 μm) on the surface of the catheter skeleton and use magnetic actuation as the second module of hybrid actuation for the robot. Here, the microiron particles (diameter: 5 μm) (Guangzhou Metallurgy Co., Ltd.) are chosen as the base material for magnetic response, and the silicone rubber, i.e., Ecoflex #20 (Beijing Angelcrete Art Landscaping Co., Ltd.), is chosen as the elastomer matrix of the composite. First, the nonmagnetized iron particles and uncured Ecoflex with a prescribed mass fraction are mixed to obtain the homogeneous ferromagnetic composite elastomer (Figure 1c‐iii). Second, the elastomer is brushed on the surface of the skeleton to form the skin via spin coating, avoiding the redundant elastomer permeating inside the skeleton (Figure 1c‐ii). After that, a uniform magnetic field is applied with the direction along the robot central axis to rearrange the iron particles, and the composite is cured in air (Figure 1c‐ii). As a result, a layer of ferromagnetic elastomer skin (thickness of ≈100–150 μm) is uniformly covered on the 3D‐printed skeleton. The optical image of the fabricated soft continuum robot is shown in Figure 1d. With an excellent breaking elongation rate of 620% and a hardness of shore of 20 A (Table S1, Supporting Information), Ecoflex grants the elastomer skin with enough softness and elasticity. In addition, the robot can obtain better actuation performance when it is subjected to an external magnetic field due to the rearrangement of the iron particles.

### 2.2 Large‐Angle Posture Control by Tendon‐Driven Robots

**Figure 2:**
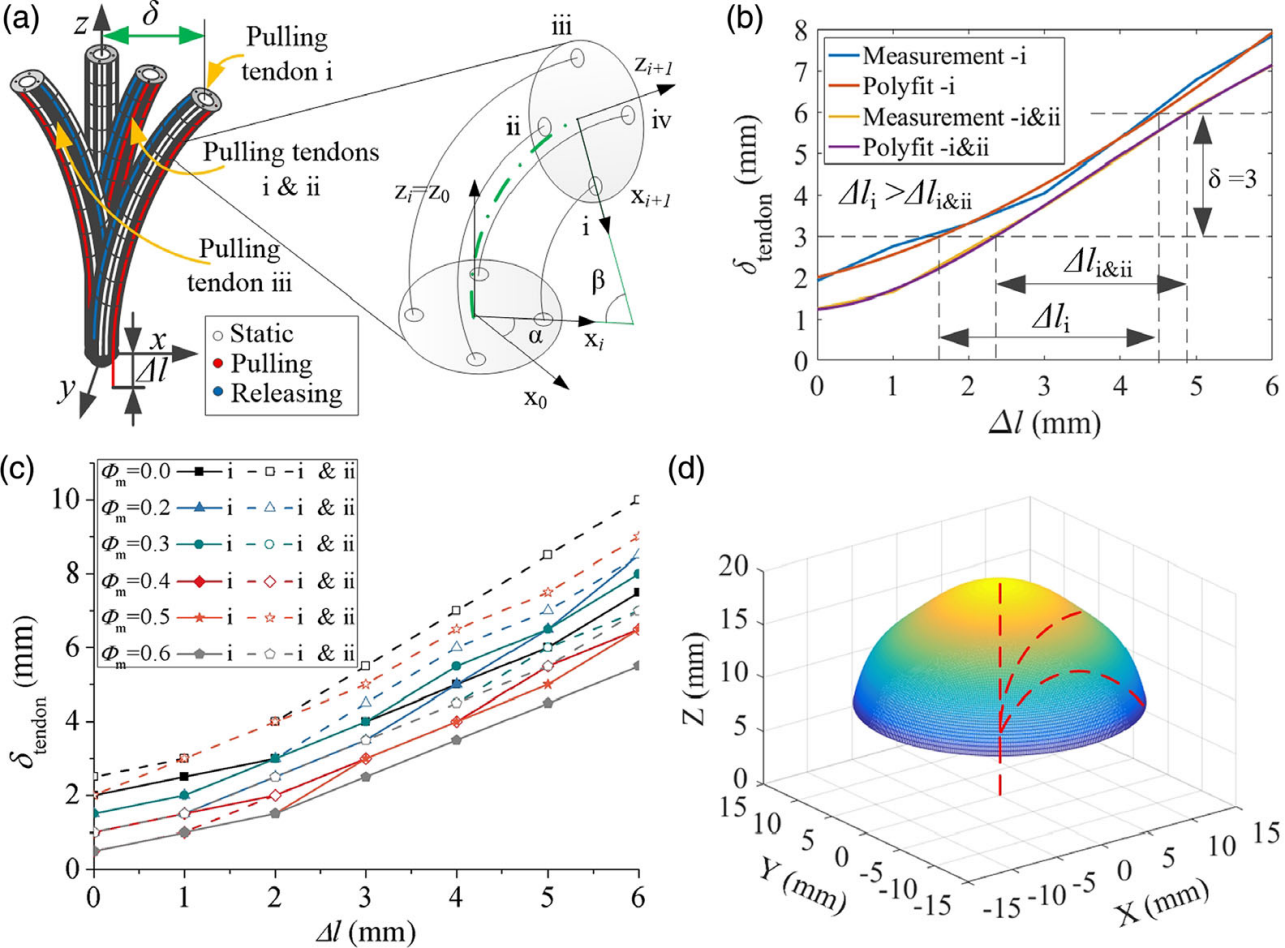
Properties of the soft continuum robot under the tendon‐driven mechanism. a) Sketch of the different postures of robots against varied tendon‐driven strategies. The enlarged part represents the theoretical model of the continuous single joint of the catheter, established using the PCCA model. The characters i, ii, iii, and iv represent the corresponding tendon. b) The deflection of catheter tip against tendon's elongation obtained from both experimental measurement and analytic curve fitting when the robot is actuated by single and adjacent pairs of tendons. The characters i, and i&ii denote the actuation strategy of the single‐pair and adjacent‐pair tendons, respectively. c) The experimental measurement of the catheter tip's deflection against tendon's elongation under different pull–release strategies for the varied mass fraction of iron particles. d) Prediction of workspace, obtained utilizing the structural symmetry of the catheter tip of the soft continuum robot.

For soft continuum robots,^[^ ^18^ ^]^ due to only one bending degree on the catheter tip, they have to rotate the entire body to achieve full‐orientation bending motion. Such kind of manipulation would result in the risk of hurting the surface of the tissue. For more friendly interaction with the tissue surface, two pairs of antagonistic tendons are designed inside the tube wall in our proposed robot. With different actuation strategies of pull–release on each pair of tendons (i, ii, iii, and iv), the robot could achieve varied postures (**Figure** **2**a), avoiding the rotation motion of the whole body.

To obtain the theoretical relationship between the posture of the catheter tip and the elongation of each tendon, we can use the piecewise constant curvature approximation (PCCA) model to separate the catheter‐active part into ten sections and establish a geometric model of continuous single joints (Figure 2a). The base coordinate system of the robot is set as {*X*_o_–*Y*_o_–*Z*_o_}. We construct the coordinate system {O_*i*_} and {O_*i*+1_} in the center of the upper plane of the *i*th and *i + *1th section, respectively. The axes of *Z* are perpendicular to the upper plane of each section, whereas the axes of *X* point to the hole of the first tendon. Besides, the *Y*‐axis of each coordinate system is determined by the right‐hand rule. Given the condition that *X*_*i*_ poses a rotation angle, i.e., *α*, with *X*_o_ along *Z*_*i*_ and that *Z*_*i*_ keeps the same direction as *Z*_o_, then, we can finally obtain the elongation of tendons for the robot as

(1) $$\left\{ \begin{array}{l} \Delta l_{1}=10r_{1}\beta \cos\alpha \\ \Delta l_{2}=10r_{1}\beta \cos(\alpha -\pi /2)\\ \Delta l_{3}=10r_{1}\beta \cos(\alpha -\pi )\\ \Delta l_{4}=10r_{1}\beta \cos(\alpha -3\pi /2) \end{array}\right.$$

where *β* represents the bending angle of each section and *r*_1_ denotes the distance between the center of the hole (passing through the first tendon) and the center of the plane.

The bending difference of catheter tip (without elastomer skin) between the actuation of the single pair (pulling i while releasing iii) and double pair (pulling i & ii while releasing iii & iv) of tendons is investigated first. The deflection against elongation is shown in Figure 2b. These two strategies perform a similar trend, which indicates that a circular trajectory perpendicular to the advancing direction could be reached through the cooperation of all four tendons with a proper pull–release strategy. In addition, to achieve the same deflection caused by the actuation of single‐pair tendons, less elongation is needed for the adjacent pairs of tendon‐driven strategies. Given two rotation angles of $\alpha_{\text{single}}=0$ and $\alpha_{\text{double}}=\pi /4$ corresponding to the actuation of the single and adjacent pairs of tendons, respectively, the theoretical elongation shall be $\Delta l_{1\text{\_single}}=10r_{1}\beta$ and $\Delta l_{1\text{\_double}}=\Delta l_{2\text{\_double}}=5\sqrt{2}r_{1}\beta$, where the consistency between the experimental results and the theoretical expression of Equation (1) is found. Furthermore, with the elastomer skin (embedded with different mass fractions of iron particles) coated on the skeleton, the similar difference between the actuation of single and adjacent pairs of tendons can also be noticed in the experimental results (Figure 2c).

In addition, according to Figure 2c, it is obvious that the slope of the curves becomes smaller with the increase in particle mass fraction, denoted by *ϕ*_m_, which suggests that more elongation would be needed to reach the same deflection. To analyze the phenomenon, we can first simplify the radial component of the pulling force, applied at the distal tip of the robot through the tendon, into a point force perpendicular to the longitudinal axis. The deflection can be further approximately equivalent to the bending of a cantilever beam under the concentrated force. Then, the maximum deflection at the tip can be expressed as

(2) $$\delta_{\text{tendon}}=\frac{1}{144}\frac{F_{\text{eq}}}{G}\frac{L^{3}}{I}$$

where *F*_eq_ denotes the simplified point force, *L* and *I* represent the length and moment of inertia for the active part of the robot, respectively, and the shear modulus is denoted as *G*. As there should be a proportional relationship between the pulling force and the elongation of tendons, it could be further derived to the simplified point force, i.e., $F_{\text{eq}}∼\Delta l$. Assuming that all catheters own the same geometric dimensions, the deflection can be stated as $\delta_{\text{tendon}}=C_{0}F_{\text{eq}}/G$. For achieving the same deflection, more elongation (bigger *F*_eq_) is needed as the particle mass fraction increases (Figure 2c). It is concluded that the shear modulus of the robot body experiences the same uprising trend as the particle mass fraction, i.e., $G∼\phi_{m}$.

For obtaining the workspace of the proposed soft continuum robot, we conduct actuation on a single pair of tendons on the robot without elastomer skin. The experimental measurements of deflection (Figure S2a, Supporting Information) and bending angle (up to about 100°) (Figure S2b, Supporting Information) of the catheter tip are obtained, indicating the excellent compliance of our robot. After the corresponding analytical model of the deflection is established, we can further predict the continuous surface in 3D space (Figure 2d) of the catheter tip utilizing the structural symmetry. Similarly, the same workspace can be achieved for robots with elastomer skin. Note that the soft continuum robot coated with elastomer skin leads to a higher shear modulus (see Figure S2c, Supporting Information for detail). The workspace proves a good steering performance of the proposed robot, suggesting that a complicated circumstance inside the human body can be adapted.

### 2.3 High‐Precision Positioning by Magnetic‐Driven Robots

With a varied mass fraction of iron particles embedded in the elastomer skin, the continuum robot would perform differently in both magnetic and mechanical properties. To obtain better actuation performance for later precise positioning under a magnetic field, an optimal design on the parameter of the mass fraction should be achieved.

**Figure 3:**
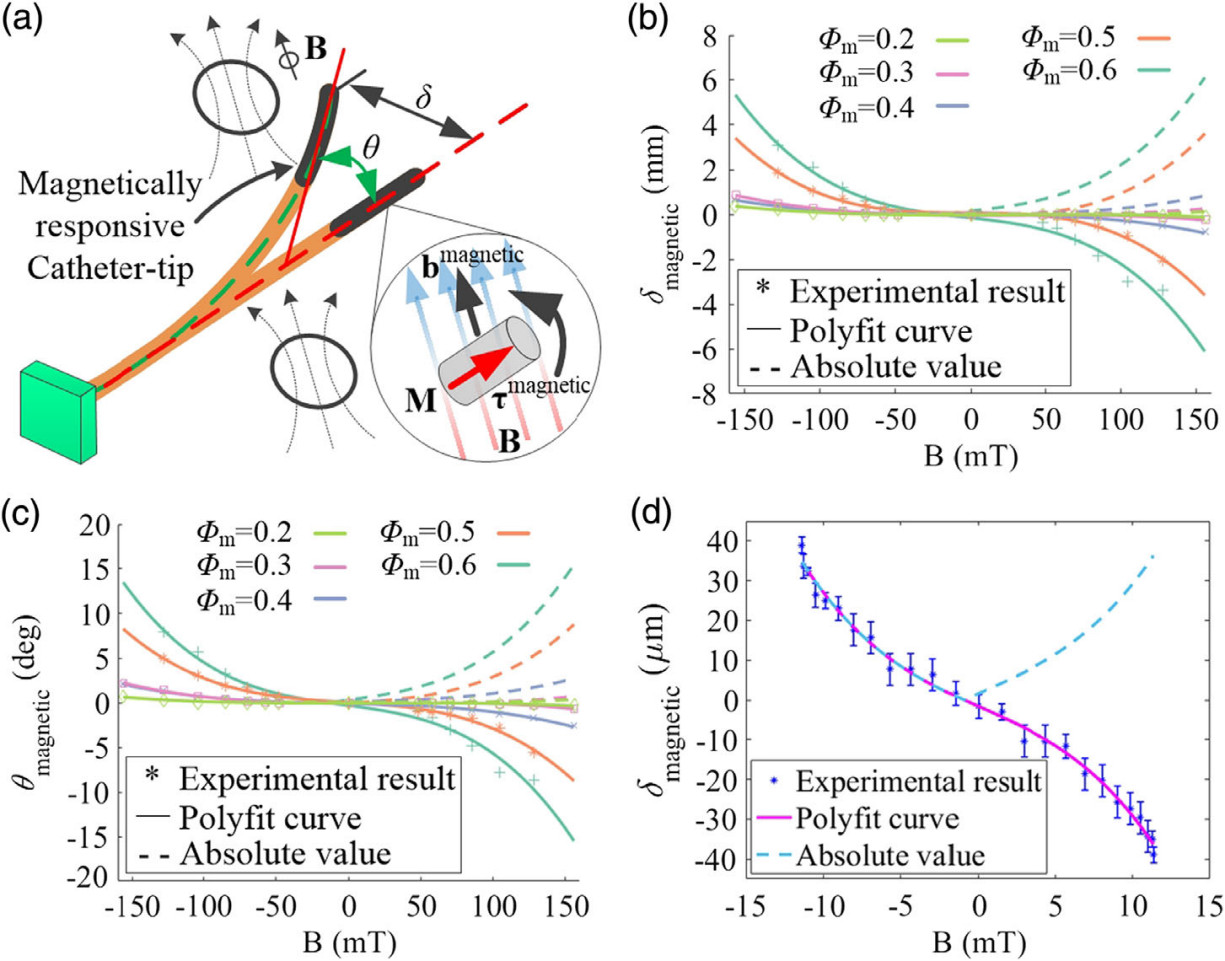
Optimal design of magnetic positioning for the soft continuum robot. a) Sketch of the proposed soft continuum robot under the actuation of an external applied magnetic field. b) Experimental measurement and the corresponding curve fitting of the deflection against the magnitude of the applied magnetic field, the catheter tip‐grown elastomer skin embedded with a different iron particles of different mass fractions. c) Experimental measurement and the corresponding curve fitting for the actuation angle against the magnitude of the applied magnetic field, the catheter tip‐grown elastomer skin embedded with different iron particles of mass fraction. d) Experimental measurement and the corresponding curve fitting for the deflection of the catheter tip actuated by a controllable magnetic field. The robot tested in this experiment owns the mass fraction of 0.6 for iron particles.

As shown in **Figure** **3**a, the volume unit within the elastomer skin bears both magnetic body torque $\tau^{\text{magnetic}}$ and magnetic body force $b^{\text{magnetic}}$, when the robot is placed under the external magnetic field. The deformation of the catheter tip will result from both the accumulated magnetic torque and force. To simplify the mechanical status of the robot, a model^[^ ^14^ ^]^ based on the magnetic Cauchy stress would be applied to conduct theoretical analysis. The applied model would cause the same deformation as that of the magnetic body torque and force. We denote the induced magnetization at any point of this continuum robot in an undeformed configuration as vector **M**, along the axial direction. The deformation gradient, deformed by the applied external magnetic field **B**, at the corresponding point, could be represented as **F**. Take the theoretical framework of ferromagnetic soft materials^[^ ^23^, ^24^ ^]^ as references, the deformation could be calculated by the magnetic Cauchy stress $\sigma^{\mathrm{magnetic}}=-B⊗FM$, where ⊗ represents the dyadic product. Assuming that the active catheter conforms to the case of small deflection when placed under a magnetic field **B** perpendicular to **M** (Figure 3a), the free end generates a deflection smaller than 10% of the length. In addition, simplifying the catheter as a uniform tube, we can reach the analytical expression for deformation of the distal tip.

(3) $$\delta_{\text{magnetic}}=\frac{16L^{3}(Do^{2}-D^{2})}{9(Do^{4}-d^{4})}\frac{\mathrm{MB}}{G}$$

where *M* and *B* represent the magnitudes of the induced magnetization of the robot and the external magnetic field, respectively. The geometry dimensions are expressed as length *L*, outer diameter *D*o, and inner diameter *d*.

As for the soft robot with ferromagnetic elastomer skin, both *M* and *G* would be affected by the mass fraction of iron particles. For the ferromagnetic particles embedded in elastomer skin, the relationship between the induced magnetization and mass fraction can be presented as $M=f(B,\phi_{m})$. According to Equation (3), the deflection caused by an external magnetic field is mainly affected by both magnitudes of the external magnetic field and its material, i.e., $\delta_{\text{magnetic}}=C_{1}(M/G)B$. For a certain mass fraction of iron particles, there should be a quadratic trend between the deflection and the magnitude of the external magnetic field, as magnetization could be approximately viewed as a linear function of the latter one before magnetic saturation.^[^ ^23^ ^]^ Given a specific external magnetic field **B**, there should be an optimal solution for the mass fraction at which a maximal deflection is obtained.

The experimental measurement and the corresponding curve fitting of deflection under a magnetic field are shown in Figure 3b. For each robot with a certain mass fraction of iron particles, the absolute value of deflection shows a similar quadratic relationship with the magnitude of the external magnetic field, in accordance with Equation (3) of the theoretical model. In addition, according to Figure 3b, the robot embedded with a higher mass fraction of iron particles generates a bigger deflection under the same magnitude of a magnetic field, especially when the mass fraction is larger than 40%. The relationship between deflection and particle mass fraction as proportional, i.e., $\delta_{\text{magnetic}}∼\phi_{m}$, can be concluded. It further suggests the material‐related variable *M*/*G* presents the same changing trend with particle mass fraction, i.e., $M/G∼\phi_{m}$. Taking the relationship between the shear modulus and mass fraction of iron particles, i.e., $G∼\phi_{m}$, into consideration, it can be concluded that the increase of induced magnetization **M** of the robot is obviously faster than that of shear modulus *G* as the mass fraction of iron particles increases. Also, the actuation angle of the robot with a higher mass fraction experiences a faster uprising for a specific external magnetic field **B** (Figure 3c), indicating the proportional relationship $\theta_{\text{magnetic}}∼\phi_{m}$. It is consistent with the trend of deflection. Moreover, the composite elastomer with a high mass fraction of iron particles (≥70%) is difficult to be coated on the robot surface (see Figure S2d, Supporting Information for detail). Therefore, the optimal mass fraction of iron particles distributed in the elastomer skin could be viewed as 60% for the proposed soft continuum robot.

With the optimal mass fraction of iron particles embedded in elastomer skin, the merit of the high‐precision positioning of the proposed soft continuum robot (the prototype is shown in Figure S1, Supporting Information) is demonstrated under a finely controllable magnetic field. According to Figure 3d, the absolute value of deflection shows an approximately quadratic relationship with the magnitude of the external magnetic field. The static positioning accuracy of the catheter tip can be achieved up to ≈2 μm, under the guidance of both the magnitude and direction of the applied magnetic field.

### 2.4 Active Steering and Positioning for Searching the Pathological Area Within the Vessel

Hereafter, the primary capabilities of steering through the complex constrained environment (tendon driven) and high‐precision positioning (magnetic actuation) of the proposed soft continuum robot are demonstrated. Moreover, additional functions granted by the functional core assembled inside the lumen of the robot are proved.

**Figure 4:**
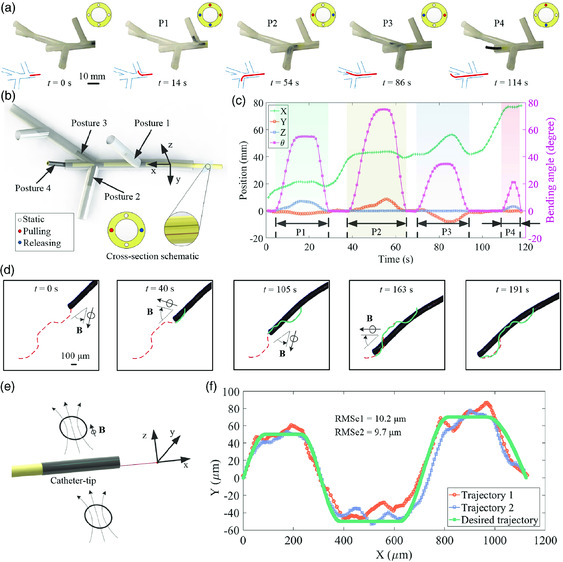
Demonstration of active steering and positioning capabilities of the soft continuum robot. a) Experimental demonstration of selectively steering within a tunnel with a series of bifurcation sections, when the robot is under the tendon‐driven mechanism. b) Schematic demonstration of different postures of the proposed soft continuum robot when passing through a branched tunnel with an active catheter tip actuated by tendons. c) Experimental measurement of the position and bending angle of the catheter tip under tendon‐driven mechanism during the entire passing period. d) The experimental process of the high‐precision positioning of the robot under actuation of the external applied magnetic field. e) Schematic demonstration of the robot positioning under the external applied controllable magnetic field. f) Experimental measurement of the trajectories of the catheter tip under magnetic actuation. The robot shows the high‐precision navigation capacity, with an external magnetic field applied at the distal magnetically responsive tip. The proximal end of the robot was pushed to advance the entire body during the whole steering and navigating process. Detailed dimensions of the demonstration setup are available in the supplemental material (Figure S4a, Supporting Information).

The skeleton of the prototype is fabricated by micro‐3D printing technology (Figure 1c‐i). The ferromagnetic composite elastomer, composed of both iron particles and Ecoflex, is coated onto the robot surface to achieve a magnetic response (Figure 1c‐ii). The experimental process of steering through a 3D branched tunnel (see Figure S4a, Supporting Information for detail) of the prototype is shown in **Figure** **4**a. Initially, the robot moves straight inside the tunnel under the push force applied at the proximal end. Upon approaching the first bifurcation, the robot is steered toward the north east by pulling both the upper and right tendons and releasing the rest of the two tendons. After accessing about 15 mm, it is retracted to the bifurcation point and keeps advancing to the second bifurcation point. The robot is actuated to access the left branch by pulling the left tendon and releasing the right one, posing an angle of 75° with the trunk. Later, the robot is retracted and performs navigation toward the right for 35° using a strategy opposite to the previous one. After another move back, the robot moves forward. Finally, the robot performs an upforward posture at about 25° by pulling the upper tendon and releasing the lower one.

The four main postures of the robot under the tendon‐driven mechanism during the steering process within a branched tunnel are shown in Figure 4b. Both the position and bending angle of the distal tip of the assembled prototype during the steering process are collected and shown in Figure 4c. According to the experimental measurement, we can easily find that there are four periods of noticeable variations of bending angles corresponding to the four postures. There are small increments along *X*‐axis and a relatively big increase along the *Z/Y* axis when the robot accesses the first two branches. A significant variation along both *X* and *Y* axes is experienced for the robot during the third branch. The incremental trend along the coordinate system agrees with the geometry contour of the experimental setup (Figure S4a, Supporting Information). The demonstration proves that our soft continuum robot has superior adaptability and navigation capability in a complex constrained environment.

The experimental process of high‐precision positioning of the proposed robot under magnetic actuation is shown in Figure 4d. During the advancing process, the robot is actuated by an external magnetic field to achieve micrometer‐scale navigation. The red dashed curve denotes the desired trajectory whereas the green curve represents the experimental trajectory of the tip. According to the two bow‐like trajectories, it can be concluded that the overall conformity is good. The schematic illustration of magnetic actuation for the robot is shown in Figure 4e, and the tip position data are shown in Figure 4f (Figure S3d, Supporting Information shown detailed magnetic field measurement). For the two experiments of positioning, the root mean square error (RMSE) for the actual trajectories relative to the desired one has been calculated as 10.2 and 9.7 μm, respectively. The tracking precision of around 10 μm grants the proposed robot's capability of conducting micromanipulation in vivo.

### 2.5 High‐Precision Manipulation for Target Therapy and Nasopharyngeal Sampling

**Figure 5:**
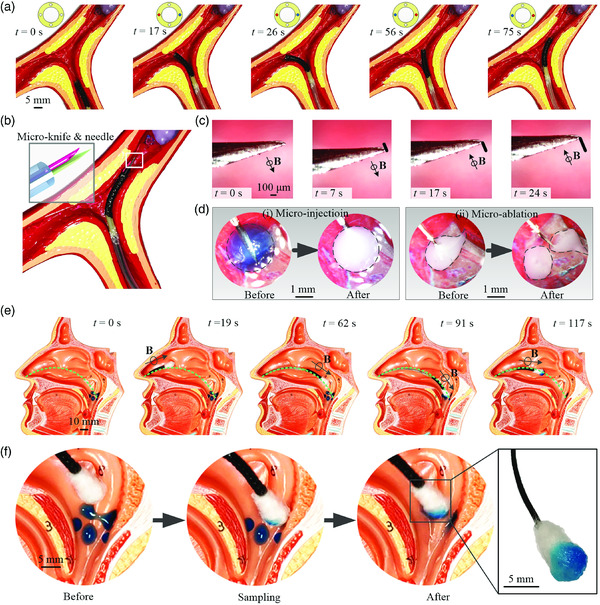
Demonstration of manipulation within the vessel model and the process of nasopharyngeal sampling. a) Demonstration process of active steering within a vessel model of the proposed soft continuum robot under the tendon‐driven mechanism. b) Schematic illustration of the soft continuum robot that approached pathology area within vessels, with a microscale knife and needle incorporated inside the robot. c) The experimental trajectory of the catheter tip under magnetic actuation. d) The potential application of micromanipulation as target therapy, including (i) microinjection and (ii) microablation. e) Experimental process of nasopharyngeal sampling within the nasal cavity, with a swab incorporated in the proposed soft continuum robot. f) The enlarged demonstration of the swab tip during the sampling process. Detailed dimensions of the demonstration setup are available in the supplemental material (Figure S4, Supporting Information).

For further extending the application of the robot, we demonstrate the additional function of high‐precision micromanipulation within a vessel model (the dimension details are shown in Figure S4b, Supporting Information) by incorporating microtools inside. The experimental process of searching the pathological area (the purple block) within the vessel model is shown in **Figure** **5**a. The proposed robot is first steered to the left vascular branch by pulling the left tendon and releasing the right one. As there is no target in this branch, the robot is retracted and actuated to the right, where the tendon‐driven strategy is opposite to the former one. After getting close to the pathological area, the microtools would protrude to conduct the corresponding micromanipulation.

The schematic illustration of the soft continuum robot that approached the pathology area within a vessel model is shown in Figure 5b. There are microscale tools, including microknife and needle, incorporated within the lumen of the robot. As a result of the magnetic response of the catheter robot, the microtools protruded will experience corresponding motion. Figure 5c shows the experimental process under magnetic actuation. The distal tip of the microknife has achieved a linear trajectory of about 300 μm under the actuation of an external magnetic field. As for the precise manipulation of the robot, it can be potentially applied to accomplish target therapy, such as target injection and target ablation (Figure 5d). The sphere‐like target experienced a visible color change after microinjection via the needle (Figure 5d‐i). Another seed‐like target has been cut into two pieces with the knife (Figure 5d‐ii). All the geometry parameters of tools, including sheath, needle, and knife, are shown in Table S2, Supporting Information.

Considering the current outbreak of COVID‐19, the potential application of nasopharyngeal sampling of the proposed soft continuum robot by incorporating a swab inside has been further demonstrated (Figure 5e). Compared with the oropharyngeal swab sampling, nasopharyngeal sampling would bring better tolerability for patients, i.e., not easily inducing coughing. It further results in a relatively long time for obtaining specimens and a lower risk of infection for medical staff.

As shown in Figure 5e, the green dashed curve passing through the middle nasal meatus represents the desired trajectory inside the nasal cavity (the dimension details are shown in Figure S4c, Supporting Information). The robot is first inserted through nostrils, along the direction parallel with the bridge of the nose. Once the swab tip reaches the middle nasal meatus, about 15 mm above the position of the snout, the robot is actuated by an external time‐varying magnetic field to achieve the probing trajectory according to the desired one. With a gentle push force applied at the proximal end of the soft continuum robot, the swab tip finally reaches the position of the nasopharynx. At this moment, the axis of the catheter tip poses about 91° with the bridge of the nose. After advancing and retracting slightly, the specimens adhered to the nasopharynx could be transferred to the surface of the swab tip. Finally, the soft continuum robot with the sampled swab can be retracted genteelly along the original inserting route. The enlarged detail of the swab tip during the sampling process is shown in Figure 5f, which demonstrates the good performance of adhering specimens.

### 2.6 Discussion

To implement surgeries within the complex constrained vessels, a small and soft body is the initial inherent requirement for continuum robots. A larger tender range‐steering ability is also needed for robots to achieve the ability to easily pass through during the period of searching for the pathological area. When conducting surgery manipulation, a higher precision and smaller dynamic error are beneficial for improving safety. Furthermore, a large inner lumen shall be granted for the robot to implement versatile manipulation by encapsulating varied surgical tools. However, the existing continuum robotic technologies encounter the difficulty of achieving the comprehensive performance of large‐angle steering and high‐precision manipulation while maintaining small‐scale size.

For conventional continuum robots, most actuation techniques own a contour dimension over 3.0 mm, e.g., tendon driven, or even with a centimeter scale, e.g., fluidic‐driven and dual‐mode actuation. Although the soft continuum robot actuated by magnetic field or smart material can be designed at a small scale, the small inner lumen less than 1.0 mm limits its functions. To obtain a larger bending angle, the fluid‐driven robots show their excellent performance (usually >150°), and the robots actuated by a magnetic field also can present the bending angle ranging from 90° to 180°. In addition, a steering angle up to 80° is reported as the bottleneck for soft robots actuated by the smart material. For positioning precision, the soft continuum robots actuated by fluid or smart material presents poor performance due to the complex control model and inherent hysteresis, respectively. Benefiting from the classic control model, many tendon‐driven robots can achieve a position precision of about 2.0 mm. The magnetic actuation is reported as the most precise actuation method for soft continuum robots and has achieved tracking error up to 0.3 mm under a controllable magnetic field.^[^ ^13^ ^]^ However, magnetic actuated robots show small resistance to an external force. To achieve comprehensive performance in vivo, tendon‐driven and magnetic actuation are combined in our proposed soft continuum robot. Thanks to 3D printing technology, the proposed robot here can be developed with an outer/inner diameter of 3.3/2.4 mm. Grant the potential of accessing smaller space and carrying varied surgical tools to implement different microinvasive surgeries. Under the actuation of tendons, the proposed robot can achieve a bending angle up to ≈100°, which is slightly improved, compared with many of the existing robots with smart material. In addition, the strong resistance capability of the tendon‐driven mechanism ensures the feasibility of navigation inside a constrained vessel for the proposed soft continuum robot. Furthermore, with the guidance of a controllable magnetic field, the demonstrated dynamic error (≈10 μm) for the proposed robot is 30‐fold improved than the state of art. Upon reaching near the pathological area, the active part can be actuated by a magnetic field to achieve highly precise positioning and manipulation.

Despite the existing error bar of static positioning ranging from 2 to 4 μm (1–2 pixels), given a more accurate measuring instrument, we believe that the capability of high‐precision manipulation would be demonstrated better. As for the demonstration process of active steering within the branched tunnel, the fluctuation shown in the measurement of tendon‐driven navigation mainly comes from the manual manipulation of pushing the robot forward. Both the instability of the cantilever (a long soft tube) and the vibration of the piezoelectric motor result in most of the dynamic tracking error (the linear tracking RMSE at about 6.3 μm without applying magnetic field, as shown in Figure S3f, Supporting Information) under magnetic actuation. As this study is the first step to demonstrate the steering and positioning capacities of the proposed soft continuum robot, a fully automated closed‐loop control system remains as the future work to improve the overall performance of the robot.

## 3 Conclusion

Here, we present a millimeter‐scale soft continuum robot with the hybrid‐actuation mode: a magnetic‐ and tendon‐driven catheter with good steering and navigation capability to adapt to the complex constrained environment while being capable of conducting high‐precision manipulation. With a unique hollow structure and a large inner lumen of 2.4 mm, the proposed robot can easily deflect along all directions away from its longitudinal axis and can encapsulate different surgical tools for varied manipulation, respectively. Under the actuation of tendons, the proposed robot can achieve a bending angle up to 100° and reach a large workspace, which does help the robot pass through a complex branched vessel. To obtain better actuation performance under an applied magnetic field, we proposed an optimization for the mass fraction of iron particles embedded in the elastomer skin of the soft continuum robot. The merits of high‐precision static positioning (≈2 μm) and dynamic tracking (RMSE of ≈10 μm) were proved under a controllable magnetic field. With a combination of tendon‐driven and magnetic actuation, the prototype demonstrated good steering within a complex constrained environment and precise tracking capability. By incorporating additional functionality of surgical tools inside, the proposed soft robot can achieve versatile manipulations, e.g., target injection and ablation within vessels, and nasopharyngeal sampling. With the merits of large‐angle steering and high‐precise manipulation, the proposed millimeter‐scale soft continuum robot will present remarkable advances in the emerging area of biomedical robotics.

## 4 Experimental Section

4.1

4.1.1

##### Materials

The photosensitive resin (HD), Ecoflex 20, and iron (Fe) particles were purchased from BMF Material Technology Inc., Beijing Angelcrete Art Landscaping Co., Ltd. (the valued distributor of Smooth‐On), and Guangzhou Metallurgy Co., Ltd., respectively. The rubber tube, stainless‐steel tendons, and silicone glue were purchased from a grocery store in China.

##### 3D Printing

The skeleton of the proposed continuum robot was printed with a microscale 3D printing system named nanoArch P140 (BMF Material Technology Inc., Shenzhen, CHINA), using a kind of photosensitive resin: HD. The mechanical parameters of this resin are shown in Table S1, Supporting Information. As shown in Figure 1c‐i, the fluidic printing material was pressured out of the nozzle to form the contour layer by layer with the planar motion of the nozzle and the vertical movement of the base platform. In addition, UV light was utilized to solidify the structure. With an outer and inner diameter of 3.0 and 2.4 mm, respectively, this catheter allowed many functional components passing through (Figure S1b, Supporting Information). In addition, the four small holes with a diameter of 150 μm constructed within the body allowed two pairs of antagonistic tendons to achieve bending degree of freedom along two vertical directions. The special hollow structure established along two perpendicular directions with width a=500μm helped both magnetic and tendon actuations obtain a better bending performance. The length of the active part was 20 mm, whereas the whole printing catheter owned a range of L0=30 mm.

##### Ferromagnetic Composite Elastomer

First of all, the nonmagnetized iron particles and uncured Ecoflex with a prescribed mass fraction were added into a beaker. Then, it was stirred for 5–6 min to achieve a homogeneously mixed status. Later, the air bubbles within the mixture produced during the stirring process were removed under a vacuum pump for ≈10 min. The evenly distributed ferromagnetic composite elastomer was obtained. All the procedures are shown in Figure 1c‐iii.

##### Coating

A rod inserted inside the skeleton was driven to spin at a certain speed for avoiding the elastomer penetrating the inner side of the catheter. The ferromagnetic composite elastomer was brushed to the surface of the catheter with a thin slice that moved along the direction perpendicular to the central axis of the rod. The whole system was kept at a relative slow rotating speed with the help of the rotating platform below after the brushing step. For achieving a uniform rearrangement of the iron particles, the coating setup was placed under an external applied uniform magnetic field for the entire period of curing (exposure to air for ≈4 h). This rearrangement helped the robot obtain better motion performance under magnetic actuation. The schematic illustration is shown in Figure 1c‐ii.

##### Prototype Assembling

After the fabrication of the skeleton, four stainless steel wires with a diameter of 100 μm were carefully threaded through the preformed holes as antagonistic tendons. Later, the composite elastomer skin was cured under a uniform magnetic field, and the outer diameter of the catheter changed to Do=3.2∼3.3 mm. Finally, a rubber tube with an outer/inner diameter of 3.0/2.5 mm was glued to the proximal end of the printed catheter via silicone glue. The assembled prototype is shown in Figure S1b, Supporting Information.

## Conflict of Interest

The authors declare no conflict of interest.

## Supporting information

Supplementary Material

Supplementary Material

Supplementary Material

Supplementary Material

Supplementary Material
