# Supplementary material for: Millimeter‐Scale Soft Continuum Robots for Large‐Angle and High‐Precision Manipulation by Hybrid Actuation
Source: Adv Intell Syst. 2020 Nov 19;3(2):2000189. doi: 10.1002/aisy.202000189 (PMC7744893; doi:10.1002/aisy.202000189)
Supplement: Supplementary file 5 — Supplementary Material [file AISY-3-0-s001.doc]

DOI: 10.1002/ ((please add manuscript number))

**Article type: Full Paper**

Millimeter-scale Soft Continuum Robot for Large Angle and High Precision Manipulation by Hybrid Actuation

Tieshan Zhang, Liu Yang, Xiong Yang, Rong Tan, Haojian Lu*, Yajing Shen*

Tieshan Zhang, Liu Yang, Xiong Yang, Rong Tan, Prof. Haojian Lu, Prof. Yajing Shen

Department of Biomedical Engineering

City University of Hong Kong

Tat Chee Avenue, Kowloon, Hong Kong, China
E-mail: haojianlu2-c@my.cityu.edu.hk, [yajishen@cityu.edu.hk](mailto:yajishen@cityu.edu.hk)

Prof. Haojian Lu

The State Key Laboratory of Industrial Control and Technology

Zhejiang University

Hangzhou 310027, China

Email: luhaojian@zju.edu.cn

Prof. Yajing Shen

Shenzhen Research Institute of City University of Hong Kong

Shenzhen 518057, China

E-mail: yajishen@cityu.edu.hk

Keywords: millimeter-scale soft continuum robot, tendon-driven, magnetic actuation, micromanipulation

Developing small-scale soft continuum robots with large-angle steering capacity and high-precision manipulation offers broad opportunities in various biomedical settings. However, existing continuum robots reach the bottleneck in actuation on account of the contradiction among small size, compliance actuation, large tender range, high precision, and small dynamic error. Here, we report a 3D printed millimeter-scale soft continuum robot with an ultrathin hollow skeleton wall (300 μm) and a large inner-to-outer ratio (0.8). After coating a thin ferromagnetic elastomer layer (~100-150 μm), the proposed soft continuum robot equipped with hybrid actuation (tendon and magnetic driven mode) can achieve large-angle (up to 100 degrees) steering and high precision (low to 2 μm for static positioning) micromanipulation simultaneously. Specifically, our robot implements an ultralow dynamic tracking error ~10 μm, which is ~30 folds improved than the state-of-art. Combined with a micro-needle/knife or nasopharyngeal swab, our robot reveals the potentials for versatile biomedical applications, such as drug injection on the target tissue, diseased tissue ablation, and COVID-19 nasopharyngeal sampling. The proposed millimeter-scale soft continuum robot presents a remarkable advance in large range and high precise actuation, which provides a new method for miniature continuum robot design and would find broad applications in biomedical engineering.

1. Introduction

Small-scale continuum robots with soft body own great application potentials^[1-3]^ on various pathological areas inside the human body (**Figure 1**a) owing to their advances in steering and navigation capacities within constrained environments. To carry out effective operations in the abovementioned areas, the contour of the robot should maintain a millimeter-scale while a large inner lumen shall be granted for carrying surgical tools. Moreover, to achieve friendly contact and easily passing through within the complex constrained environment (e.g., blood vessels) (**Figure 1**b), inherent compliance and larger tender range are essentially needed for the robot when searching for pathological areas. Besides, the merits of higher precision and smaller dynamic error are also necessarily required for the robot to implement a safer and more effective target therapy in vivo.

To control the continuum robots to follow the desired trajectories, multiple actuation mechanisms have been developed. Tendon-driven robots,^[4-6]^ with relatively rigid main body actuated via pairs of cables, have appealed to favor of many scholars. Based on the multi-sectional tubular structure, tendon-driven robots can be easily established analytical kinematic expressions and utilized for delivering smaller surgery tools, which benefits the posture prediction and multitasking operation in endoscopic surgical procedures. This type of actuation can usually reach a steering angle of ~100 degrees and achieve the positioning precision of around 2.0 mm. However, traditional tendon-driven continuum robots have the difficulty of scaling down to a small scale while keeping a large inner lumen through conventional manufacturing technology. For reducing the components and rigidity of the end-effector and maintaining a large workspace simultaneously, the fluid-driven mechanism^[7-9]^ has been proposed as a substitute. Although the soft body grants the fluidic actuation robots with the merits of friendly contact and excellent bending angle (>150 degrees), the complexity of the control system keeps increasing^[10]^ due to the nonlinear properties. Therefore, precise positioning is hard to achieve. Besides, the fluid-driven robots remain the contour even larger than the tendon-driven ones.

For achieving a small-scale contour, numerous advanced materials have been proposed and demonstrate unique superiority^[11, 12]^. Specifically, soft continuum robots embedded with tiny magnets^[13-15]^ or fabricated via ferromagnetic composite^[16]^ demonstrate precise steering capability (bending angle range from 90 to 180 degrees, and positioning precision up to 0.3 mm ^[13]^) under an external controllable magnetic field. Usually, the magnetic actuated soft continuum robots’ diameter are smaller than 3.0 mm, which ensure their capabilities of endoscopic imaging inside bronchus or target laser ablation inside a cerebrovascular phantom network.However, magnetic actuated soft continuum robots are hard to keep steady when suffered from an external force, e.g., the resistance from the channel wall. Morevoer, the proposed robot body without inner lumen limits the robots’ function integration. Besides, the tiny rigid magnet tip suffers the risk of breaking off in vivo during the manipulation. To achieve safer and direct actuation, shape-memory materials^[17, 18]^ have been discovered and widely applied as actuators for continuum soft robots to accomplish specific tasks, i.e., cardiovascular inspection, and nasopharyngeal drug delivery. The self-deformation material can achieve actuation with the whole body while maintaining a small-scale in the meantime. However, it is hard to realize quick response and precise positioning simultaneously due to the inherent hysteresis phenomenon. Although shape-memory material actuated soft continuum robots have potentials to be developed down to millimeter scale, the large steering angle is still remains challenges.

To achieve precise posture prediction and variable stiffness against different environments, scholars developed some manipulators^[19, 20]^ combining pneumatic and tendons. This type of hybrid-actuation robot demonstrates the features of friendly gripping through mimicking the octopus’ muscles and performs the bending angle larger than 90 degrees. Whereas, it remains shortcomings, such as the relative large scale size, low static and dynamic precision, and the complex control strategy. To improve compliance and decrease the complexity of the actuation system, a shape memory polymer-based fluid actuated arm^[21]^ was proposed to be applied in minimally invasive surgery (MIS). Although the actuation is reported to be able to achieve helical bending (~140 degrees) and variable stiffness, the properties of centimeter-scale contour and thermal hysteresis exist.

Considering the circumstances that most of the existing continuum robots are hard to achieve a comprehensive performance of large-angle steering and high precision manipulation while maintaining small-scale size, here, we propose a millimeter-scale soft continuum robot with hybrid-actuation mode to tackle the problem (**Figure 1**b). Tendon-driven mechanisms could achieve relatively large angle steering control and resist higher external forces for searching pathological areas in vivo. A precisely controlled external magnetic field is capable of leading the high precision manipulation after arrival. With the help of micro 3D printing technology, the skeleton with an outer/inner diameter of 3.0/2.4 mm could be fabricated precisely (**Figure 1**c-i). Four through-holes (diameter of 150 μm) can also be constructed within the ultra-thin wall (300 μm). To integrate magnetic actuation and increase compliance, we grow an elastomer skin layer (thickness of ~100-150 μm) embedded with iron particles onto the surface of the robot, shown as **Figure 1**c-ii. With proper pull-release strategies of tendons, the robot can achieve a bending angle up to 100 degrees and reach a large workspace. Under the actuation of the magnetic field, the precision of static positioning low to 2 μm and a low dynamic tracking error ~ 10 μm are finally achieved. With a combination of both tendon-driven and magnetic actuation, the proposed soft continuum robot is demonstrated the excellent capability of steering within the vessel-mimic branched tunnel and precise tracking. We further demonstrate the versatile micromanipulation capabilities through equipping the microtools to present more potential applications with relevance to clinical surgery, e.g., microinjection/ablation, nasopharyngeal sampling.

2. Results and discussion

2.1. Hybrid actuation millimeter-scale soft continuum robot design

To obtain a large bending angle and predict the posture accurately, a tendon-driven mechanism is employed as the actuation for the robot during the period of navigating to the pathological area. For the sake of achieving the steering capacity along the plane perpendicular to its central axis, two pairs of antagonistic tendons are applied. Four through-holes are constructed within the wall to implement the tendons (stainless steel wire with a diameter of 100 μm), placed at 90 degrees between each other. To adapt to the circumstances within vessels in vivo, the outer diameter of the skeleton is designed as 3.0 mm, and a hollow structure (**Figure S1**a) along the bending directions is designed to increase the compliance of the robot. Furthermore, the designed large inner lumen promotes the robot to explore the versatile capacities via encapsulating varied surgical tools.

Here, the micro 3D printing system (nanoArch P140, BMF Material Technology Inc.) with a printing precision of 10 μm is employed to fabricate such a delicate robot skeleton (**Figure 1**c-i). For obtaining enough strength and elasticity of the structure, a type of ductile photosensitive resin (HD, BMF Material Technology Inc.) with an elastic modulus of 3.6 GPa is utilized as the printing material. As shown in **Table S1**, the material possesses the breaking elongation rate of 24.3%, leading to a low risk of breaking off for large deformation. The skeleton is printed with an outer/inner diameter of $D/d=3.0/2.4$ mm, hollow section $a=500$μm, through-holes $d_{h}=150$ μm (**Figure S1**b). As a result, an ultrathin wall of 300 μm is obtained, which is the thinnest wall among all reported continuum tendon-driven robot^[22]^. Moreover, the large inner-to-outer ratio, i.e., 0.8, grants the robot high superiority in encapsulating versatile small surgical tools while maintaining a tiny size (**Figure 1**b). Meanwhile, the hollow skeleton allows the robot to easily achieve a large bending motion while keeping enough stiffness.

To achieve the high-precise manipulation and maintain friendly interaction with tissue, we decorate a thin layer of ferromagnetic composite elastomer skin (~100-150 μm) on the surface of the catheter skeleton and employ the magnetic actuation as the second module of hybrid-actuation for the robot. Here, the micro iron particles (diameter: 5 μm) (Guangzhou Metallurgy Co., Ltd.) are chosen as the base material for the magnetic response, and the silicone rubber, i.e., Ecoflex #20 (Beijing Angelcrete Art Landscaping Co., Ltd.), is chosen as the elastomer matrix of the composite. Firstly, the non-magnetized iron particles and uncured Ecoflex with a prescribed mass fraction are mixed to obtain the homogeneous ferromagnetic composite elastomer (**Figure 1**c-iii). Secondly, the elastomer is brushed on the surface of the skeleton to form the skin via spin coating, avoiding the reduntant elastomer permeating inside theskeleton (**Figure 1**c-ii). After that, a uniform magnetic field is applied with the direction along the robot central axis to rearrange the iron particles, and the composite is cured in the air (**Figure 1**c-ii). As a result, a layer of ferromagnetic elastomer skin (thickness of ~100 to 150 μm) is uniformly covered on the 3D printed skeleton. The optical image of the fabricated soft continuum robot is shown in **Figure 1**d. With an excellent breaking elongation rate of 620 % and a hardness of shore 20 A (**Table S1**), the Ecoflex grants the elastomer skin with enough softness and elasticity. Besides, the robot can obtain a better actuation performance when it is subjected to an external magnetic field due to the rearrangement of the iron particles.

2.2. Large-angle posture control by tendon-driven

For the soft continuum robots^[18]^ owning only one bending degree on the catheter-tip, they have to rotate the entire body to achieve the full-orientation bending motion. Such kind of manipulation would result in the risk of hurting the surface of the tissue. For more friendly interaction with the tissue surface, two pairs of antagonistic tendons are designed inside the tube wall in our proposed robot. With different actuation strategies of pull-release on each pair of tendons (ⅰ, ⅱ, ⅲ, and ⅳ), the robot could achieve varied postures (**Figure 2**a), avoiding the rotation motion of the whole body.

To obtain the theoretical relationship between the posture of catheter-tip and the elongation of each tendon, we can use the piecewise constant curvature approximation (PCCA) model to separate the catheter active part into 10 sections and establish a geometric model of continuous single-joint (**Figure 2**a). The base coordinate system of the robot is set as {X*_o_*-Y*_o_*-Z*_o_*}. We construct the coordinate system {O*_i_*} and {O*_i+1_*} in the center of the upper plane of the *i*th and *i+1*th section, respectively. The axes of Z are perpendicular to the upper plane of each section, while the axes of X point to the hole of the first drive tendon. Besides, the Y-axis of each coordinate system is determined by the right-hand rule. Given the condition that the X*_i_* poses a rotation angle, i.e., *α*, with X*_o_* along Z*_i_* and that Z*_i_* keeps the same direction as Z*_o_*. Then, we can finally obtain the elongation of tendons for the robot as

$\left\{\begin{matrix} \triangle l_{1}=10r_{1}\beta\cos\alpha\\ \triangle l_{2}=10r_{1}\beta\cos(\alpha-\pi/2) \\ \triangle l_{3}=10r_{1}\beta\cos(\alpha-\pi) \\ \triangle l_{4}=10r_{1}\beta\cos(\alpha-3\pi/2) \end{matrix}\right.$ (1)

where *β* represents the bending angle of each section, *r*_1_ denotes the distance between the center of the hole (passing through the first tendon) and the center of the plane.

The bending difference of catheter-tip (without elastomer skin) between the actuation of single-pair (pulling ⅰ while releasing ⅲ) and double-pair (pulling ⅰ & ⅱ while releasing ⅲ & ⅳ) of tendons is investigated first. The deflection against elongation is shown in **Figure 2**b. These two strategies perform a similar trend, which indicates that a circular trajectory perpendicular to the advancing direction could be reached through the cooperation of all four tendons with a proper pull-release strategy. Besides, to achieve the same deflection caused by the actuation of single-pair tendons, less elongation is needed for the adjacent pairs of tendon-driven strategy. Given two rotation angles of $\alpha_{\text{single}}\text{=}0$ and $\alpha_{\text{double}}\text{=}\pi/4$ corresponding to the actuation of single and adjacent pairs of tendons, respectively, the theoretical elongation shall be $\Delta l_{\text{1\_single}}\text{=1}0r_{1}\beta$ and $\Delta l_{\text{1\_double}}\text{=}\Delta l_{\text{2\_double}}\text{=}5\sqrt{2}r_{1}\beta$, where the consistency between the experimental results and the theoretical expression of **Equation 1** would be found. Furthermore, with the elastomer skin (embedded with different mass fractions of iron particles) coated on the skeleton, the similar difference between the actuation of single and adjacent pairs tendons can also be noticed in the experimental results (**Figure 2**c).

Besides, according to **Figure 2**c, it is obvious that the slope of curves becomes smaller with the increase of particle mass fraction, denoted by *ϕ*_m_, which suggests more elongation would be needed to reach the same deflection. To analyze the phenomenon, we can firstly simplify the radial component of pulling force, applied at the distal tip of the robot through the tendon, into a point force perpendicular to the longitudinal axis. The deflection can be further approximately equivalent to the bending of a cantilever beam under the concentrated force. Then, the maximum deflection at the tip can be expressed as

$\delta_{\text{tendon}}=\frac{1}{144}\frac{F_{\text{eq}}}{G}\frac{L^{3}}{I}$ (2)

where *F*_eq_ denotes the simplified point force, *L* and *I* represent the length and moment of inertia for the active part of the robot, respectively, and the shear modulus is denoted as *G*. As there should be a proportional relationship between the pulling force and the elongation of tendons, it could be further derived to the simplified point force, i.e. $F_{\text{eq}}\sim\triangle l$. Assuming that all catheters own the same geometric dimensions, the deflection can be stated as $\delta_{\text{tendon}}=C_{0}F_{\text{eq}}/G$. For achieving the same deflection, more elongation (bigger *F*_eq_) is needed as the particle mass fraction increasing (**Figure 2**c). It is concluded that the shear modulus of the robot body experiences the same up-rising trend as the particle mass fraction, i.e. .

For obtaining the workspace of the proposed soft continuum robot, we conduct actuation on a single pair of tendons on the robot without elastomer skin. The experimental measurements of deflection (**Figure S**2a) and bending angle (up to about 100 degrees) (**Figure S**2b) of the catheter-tip are obtained, indicating the excellent compliance of our robot. After the corresponding analytical model of the deflection being established, we can further predict the continuous surface in 3-D space (**Figure 2**d) of the catheter-tip by utilizing the structural symmetry. Similarly, the same workspace can be achieved for robots with elastomer skin. Note that the soft continuum robot coated with elastomer skin leads to a higher shear modulus (see **Figure S2**c in the supplementary material for detail). The workspace proves a good steering performance of the proposed robot, suggesting a complicated circumstance inside the human body can be adapted.

**2.3. High-precision positioning by magnetic-driven**

With a varied mass fraction of iron particles embedded in the elastomer skin, the continuum robot would perform differently in both magnetic and mechanical properties. To obtain better actuation performance for later precise positioning under the magnetic field, an optimal design on the parameter of the mass fraction should be achieved.

As depicted in **Figure 3**a, the volume unit within the elastomer skin bears both magnetic body torque $\mathbf{\tau}^{\text{magnetic}}$and magnetic body force $\mathbf{b}^{\text{magnetic}}$, when the robot is placed under the external magnetic field. The deformation of the catheter-tip will be resulted from both the accumulated magnetic torque and force. To simplify the mechanical status of the robot, a model ^[14]^ based on the magnetic Cauchy stress would be applied to carry out the theoretical analysis. The applied model would cause the same deformation as that of magnetic body torque and force. We denote the induced magnetization at any point of this continuum robot in an undeformed configuration as a vector **M**, along the axial direction. The deformation gradient, deformed by applied external magnetic field **B**, at the corresponding point could be represented as **F**. Take the theoretical framework of ferromagnetic soft materials^[23, 24]^ as references, the deformation could be calculated by the magnetic Cauchy stress $\mathbf{\sigma}^{magnetic}=-\mathbf{B}\otimes\mathbf{FM}$, whererepresents the dyadic product. Assuming the active catheter conforms to the case of small deflection when placed under a magnetic field **B** perpendicular to **M** (**Figure 3**a), meaning the free end generates a deflection smaller than 10% of the length. Besides, simplifying the catheter as a uniform tube, we can reach the analytical expression for the deformation of the distal tip as below:

$\delta_{\text{magnetic}}=\frac{16L^{3}(Do^{2}-D^{2})}{9(Do^{4}-d^{4})}\frac{MB}{G}$ (3)

where *M* and *B* represent the magnitudes of the induced magnetization of the robot and the external magnetic field, respectively. The geometry dimensions are expressed as length *L*, outer diameter *Do*, and inner diameter *d*.

As for the soft robot with ferromagnetic elastomer skin, both *M* and *G* would be affected by the mass fraction of iron particles. For the ferromagnetic particles embedded in elastomer skin, the relationship between the induced magnetization and the mass fraction can be presented as. According to **Equation** **3**, the deflection caused by an external magnetic field is mainly affected by both magnitudes of the external magnetic field and its material, i.e. $\delta_{\text{magnetic}}\text{=}C_{1}(M/G)B$. For a certain mass fraction of iron particles, there should be a quadratic trend between the deflection and the magnitude of the external magnetic field, since the magnetization could be approximately viewed as a linear function of the latter one before magnetic saturation.^[23]^ Given a specific external magnetic field *B*, there should be an optimal solution for the mass fraction at which a maximal deflection is obtained.

The experimental measurement and the corresponding curve-fitting of deflection under a magnetic field are shown in **Figure 3**b. For each robot with a certain mass fraction of iron particles, the absolute value of the deflection shows a similar quadratic relationship with the magnitude of the external magnetic field, which is in accordance with **Equation 3** of the theoretical model. Besides, according to **Figure 3**b, the robot embedded with a higher mass fraction of iron particles generates a bigger deflection under the same magnitude of a magnetic field, especially when the mass fraction is larger than 40%. The relationship between deflection and particle mass fraction as proportional, i.e. , can be concluded. It further suggests the material-related variable *M* /*G* presents the same changing trend with particle mass fraction, i.e. . Taking the relationship between the shear modulus and mass fraction of iron particles, i.e. , into consideration, it can be concluded that the increase of induced magnetization *M* of the robot is obviously faster than that of shear modulus *G* as the increasing of mass fraction of iron particles. Also, the actuation angle of the robot with a higher mass fraction experiences a faster up-rising for a specific external magnetic field *B* (**Figure 3**c), indicating the proportional relationship . It is consistent with the trend of deflection. Moreover, the composite elastomer with a high mass fraction of iron particles (≥70%) is difficult to coat on the robot surface (see **Figure S2**d in the supplementary material for detail). Therefore, the optimal mass fraction of iron particles distributed in the elastomer skin could be viewed as 60% for the proposed soft continuum robot.

With the optimal mass fraction of iron particles embedded in elastomer skin, the merit of high-precision positioning of the proposed soft continuum robot (the prototype is shown in **Figure S**1) is demonstrated under a finely controllable magnetic field. According to **Figure 3**d, the absolute value of the deflection shows an approximately quadratic relationship with the magnitude of the external magnetic field. The static positioning accuracy of the catheter-tip can be achieved up to ~2μm, under the guidance of both magnitude and direction of the applied magnetic field.

**2.4. Active steering and positioning for searching pathological area within the vessel**

Hereafter, the primary capabilities of steering through the complex constrained environment (tendon-driven) and high-precision positioning (magnetic actuation) of the proposed soft continuum robot are demonstrated. Moreover, additional functions granted by the functional core assembled inside the lumen of the robot are proved.

The skeleton of the prototype is fabricated by micro-3D printing technology (**Figure 1**c-ⅰ). The ferromagnetic composite elastomer, composed of both iron particles and Ecoflex, is coated onto the robot surface to achieve a magnetic response (**Figure 1**c-ⅱ). The experimental process of steering through a three-dimensional branched tunnel (see **Figure S4**a for detail) of the prototype is illustrated in **Figure 4**a. Initially, the robot moves straight forward inside the tunnel under the push force applied at the proximal end. Upon approaching the first bifurcation, the robot is steered toward north-east by pulling both the upper and right tendons and releasing the rest two tendons. After accessing about 15 mm, it is retracted to the bifurcation point and keep advancing to the second bifurcation point. The robot is actuated to access the left branch by pulling the left tendon and releasing the right one, posing an angle of 75 degrees with the trunk. Later, the robot is retracted and performs the navigation towards the right for 35 degrees by using a strategy opposite to the previous one. After another move back, the robot moves forward straightly. Finally, the robot performs an up-forward posture about 25 degrees though pulling the upper tendon and releasing the lower one.

The four main postures of the robot under the tendon-driven mechanism during the steering process within a branched tunnel are demonstrated in **Figure 4**b. Both the position and bending angle of the distal tip of the assembled prototype during the steering process are collected and presented in **Figure 4**c. According to the experimental measurement, we can easily find that there are four periods of noticeable variation of bending angle correspond to the four postures. There are small increments along X-axis and a relatively big increase along the Z/Y axis when the robot is accessing the first two branches. A significant variation along both X and Y axes is experienced for the robot during the third branch. The incremental trend along the coordinate system agrees with the geometry contour of the experimental setup (**Figure S4**a). The demonstration proves that our soft continuum robot has superior adaptability and navigation capability in a complex constrained environment.

The experimental process of high-precision positioning of the proposed robot under magnetic actuation is shown in **Figure 4**d. During the advancing process, the robot is actuated by an external magnetic field to achieve micrometer-scale navigation. The red dashed curve denotes the desired trajectory while the green curve represents the experimental trajectory of the tip. According to the two bow-like trajectories, it can be concluded that the overall conformity is good. The schematic illustration of magnetic actuation for the robot is presented in **Figure 4**e, and the tip position data are depicted in **Figure 4**f (**Figure S3**d presents detailed magnetic field measurement). For the two experiments of positioning, the Root Mean Square Error (RMSE) for the actual trajectories relative to the desired one has been calculated as 10.2 and 9.7 μm, respectively. The tracking precision of around 10 μm grants the proposed robot’s capability of carrying out micromanipulation in vivo.

**2.5. High-precision manipulation for target therapy and nasopharyngeal sampling**

For further extending the application of the robot, we demonstrate the additional function of high-precision micromanipulation within a vessel model (the dimension details are shown in **Figure S4**b) by incorporating micro-tools inside. The experimental process of searching the pathological area (the purple block) within the vessel model is presented in **Figure 5**a. The proposed robot is firstly steered to the left vascular branch by pulling the left tendon and releasing the right one. Since there is no target in this branch, the robot is retracted and actuated to the right, where the tendon-driven strategy is opposite to the former one. After getting close to the pathological area, the micro-tools would protrude to carry out the corresponding micromanipulation.

The schematic illustration of the soft continuum robot approached the pathology area within a vessel model is presented in **Figure 5**b. There are micro-scale tools, including micro-knife and needle, incorporated within the lumen of the robot. As a result of the magnetic response of the catheter robot, the micro-tools protruded will experience corresponding motion. **Figure 5**c illustrates the experimental process under magnetic actuation. The distal tip of the micro-knife has achieved a linear trajectory of about 300 μm under the actuation of an external magnetic field. As for the precise manipulation of the robot, it can be potentially applied to accomplish target therapy, such as target injection and target ablation (**Figure 5**d). The sphere-like target has experienced a visible color change after micro-injection via the needle (**Figure 5**d-i). Another seed-like target has been cut into two pieces with the knife (**Figure 5**d-ii). All the geometry parameters of tools, including sheath, needle, and knife, are shown in **Table S2** in the supplemental material.

Considering the current outbreak of COVID-19, the potential application of nasopharyngeal sampling of the proposed soft continuum robot by incorporating a swab inside has been further demonstrated (**Figure 5**e). Compared with the oropharyngeal swab sampling, nasopharyngeal sampling would bring better tolerability for patients, i.e., not easily inducing coughing. It further results in a relatively long time for obtaining specimens and a lower risk of infection for medical staff.

As shown in **Figure 5**e, the green dashed curve passing through the middle nasal meatus represents the desired trajectory inside the nasal cavity (the dimension details are shown in **Figure S4**c). The robot is firstly inserted through nostrils, along the direction parallel with the bridge of the nose. Once the swab-tip reaching the middle nasal meatus, about 15 mm above the position of the snout, the robot is actuated by an external time-varying magnetic field to achieve the probing trajectory according to the desired one. With a gentle push force applied at the proximal end of the soft continuum robot, the swab-tip finally reaches the position of the nasopharynx. At this moment, the axis of the catheter-tip poses about 91 degrees with the bridge of the nose. After advancing and retracting slightly, the specimens adhered to the nasopharynx could be transferred to the surface of the swab-tip. Finally, the soft continuum robot with the sampled swab can be retracted genteelly along the original inserting route. The enlarged detail of the swab-tip during the sampling process is illustrated in **Figure 5**f, which demonstrates the good performance of adhering specimens.

**2.6. Discussion**

To implement surgeries within the complex constrained vessels, small and soft body is the initially inherent requirements for the continuum robots. A larger tender range steering ability is also needed for the robots to achieve easily passing through during the period of searching for the pathological area. When carrying out the surgery manipulation, higher precision and smaller dynamic error are beneficial to improving the safety. Furthermore, a large inner lumen shall be granted for the robot to implement versatile manipulation by encapsulating varied surgical tools. However, existing continuum robotic technologies encounter the difficulty of achieving a comprehensive performance of large-angle steering and high precision manipulation while maintaining small-scale size.

For conventional continuum robots, most actuation techniques own a contour dimension over 3.0 mm, e.g., tendon driven, or even with a centimeter scale, e.g., fluidic driven and dual-mode actuation. Although the soft continuum robot actuated by magnetic field or smart material can be designed at small-scale, the small inner lumen less than 1.0 mm limits their functions. To obtain a larger bending angle, the fluid-driven robots show their excellent performance (usually >150 degrees), and the robots actuated by a magnetic field also can present the bending angle range from 90 to 180 degrees. Besides, a steering angle up to 80 degrees is reported as the botleneck for the soft robots actuated by the smart material. For positioning precision, the soft continuum robots actuated by fluid or smart material presents poor performance due to the complex control model and the inherent hysteresis, respectively. Benefit from the classic control model, many tendon-driven robots can achieve position precision about 2.0 mm. The magnetic actuation is reported as the most precise actuation method for soft continuum robots, and has achieved tracking error up to 0.3 mm under a controllable magnetic field.^[13]^ However, magnetic actuated robots show small resistance to an external force. To achieve comprehensive performance in vivo, tendon-driven and magnetic actuation are combined in our proposed soft continuum robot. Thanks to the 3D printing technology, the proposed robot here can be developed with an outer/inner diameter of 3.3/2.4 mm. Grant the potential of accessing smaller space and carrying varied surgical tools to implement different micro-invasive surgeries. Under the actuation of tendons, the proposed robot can achieve a bending angle up to ~ 100 degrees, which is slightly improved compared with many of the existing robots with smart material. Besides, the strong resistance capability of the tendon-driven mechanism ensures the feasibility of navigation inside a constrained vessel for the proposed soft continuum robot. Furthermore, with the guidance of a controllable magnetic field, the demonstrated dynamic error (~ 10 μm) for the proposed robot is 30 folds improved than the state-of-art. Upon reaching near the pathological area, the active part can be actuated by a magnetic field to achieve highly precise positioning and manipulation.

Despite the existing error bar of static positioning ranges from 2 to 4 μm (1 to 2 pixels), given a more accurate measuring instrument, we believe that the capability of high-precision manipulation would be demonstrated better. As for the demonstration process of active steering within the branched tunnel, the fluctuation showed in the measurement of tendon-driven navigation mainly comes from the manual manipulation of pushing the robot forward. Both the instability of cantilever (a long soft tube) and the vibration of piezoelectric motor result in most of the dynamic tracking error (the linear tracking RMSE about 6.3 μm without being applied magnetic field, shown as **Figure S3**f) under magnetic actuation. As this study is the first step to demonstrate the steering and positioning capacities of the proposed soft continuum robot, a fully-automated closed-loop control system remains as future work to improve the overall performance of the robot.

3. Conclusion

Here, we present a millimeter-scale soft continuum robot with hybrid-actuation mode: magnetic and tendon driven catheter with good steering and navigation capability to adapt complex constrained environment while capable of carrying out high-precision manipulation. With a unique hollow structure and a large inner lumen of 2.4 mm, the proposed robot can easily deflect along all directions away from its longitudinal axis and can encapsulate different surgical tools for varied manipulation, respectively. Under the actuation of tendons, the proposed robot can achieve a bending angle up to 100 degrees and reach a large workspace, which does help the robot to pass through a complex branched vessel. To obtain better actuation performance under an applied magnetic field, we proposed an optimization for the mass fraction of iron particles embedded in the elastomer skin of the soft continuum robot. The merits of high-precision static positioning (low to ~ 2 μm) and dynamic tracking (low RMSE to ~ 10 μm) were proved under a controllable magnetic field. With a combination of tendon-driven and magnetic actuation, the prototype was demonstrated good steering within a complex constrained environment and precise tracking capability. By incorporating additional functionality of surgical tools inside, the proposed soft robot can achieve versatile manipulations, e.g., target injection and ablation within vessels, and nasopharyngeal sampling. With the merits of large-angle steering and high-precise manipulation, the proposed millimeter-scale soft continuum robot will present a remarkable advance in the emerging area of biomedical robotics.

4. Experimental Section

*Materials*: The photosensitive resin (HD), Ecoflex 20, and iron (Fe) particles were purchased from BMF Material Technology Inc., Beijing Angelcrete Art Landscaping Co., Ltd. (the valued distributor of Smooth-On), and Guangzhou Metallurgy Co., Ltd., respectively. The rubber tube, stainless-steel tendons, and silicone glue were purchased from a grocery store in China.

*3D printing*: The skeleton of the proposed continuum robot is printed with a micro-scale 3D printing system named nanoArch P140 (BMF Material Technology Inc., Shenzhen, CHINA) using a kind of photosensitive resin: HD. The mechanical parameters of this resin are shown in **Table S1** in the supplemental material. As shown in **Figure 1**c-ⅰ, the fluidic printing material is pressured out of the nozzle to form the contour layer by layer with the planar motion of the nozzle and the vertical moving of the base platform. Besides, a UV light is utilized to solidify the structure. With an outer and inner diameter of 3.0 mm and 2.4 mm, respectively, this catheter could allow many functional components passing through (**Figure S1**b). Besides, the four small holes with a diameter of 150 μm constructed within the body allow two pairs of antagonistic tendons to achieve bending degree-of-freedom along two vertical directions. The special hollow structure established along two perpendicular directions with width $a=500$μm would do help to both magnetic and tendon actuation to obtain a better bending performance. The length of the active part is 20 mm, while the whole printing catheter owns a range of $L0=30$mm.

*Ferromagnetic composite elastomer*: First of all, the non-magnetized iron particles and uncured Ecoflex with a prescribed mass fraction are added into a beaker. Then, it would be stirred for 5-6 minutes to achieve a homogeneously mixing status. Later, the air bubbles within the mixture produced during the stirring process would be removed under a vacuum pump for ~10 minutes. The evenly distributed ferromagnetic composite elastomer is obtained. All the procedures are illustrated in **Figure 1**c-ⅲ.

*Coating*: A rod inserted inside the skeleton is driven to spin at a certain speed for avoiding the elastomer penetrating the inner side of the catheter. The ferromagnetic composite elastomer is brushed to the surface of the catheter with a thin slice that moved along the direction perpendicular to the central axis of the rod. The whole system would keep a relative slow rotating speed with the help of the rotating platform below after the brushing step. For achieving a uniform rearrangement of the iron particles, the coating setup would be placed under an external applied uniform magnetic field for the entire period of curing (exposure to air for ~4 hours). This rearrangement helps the robot to obtain better motion performance under magnetic actuation. The schematic illustration is shown in **Figure 1**c-ⅱ.

*Prototype assembling*: After the fabrication of the skeleton, four stainless-steel wires with a diameter of 100 μm are carefully threaded through the preformed holes as the antagonistic tendons. Later, the composite elastomer skin is cured under the uniform magnetic field, and the outer diameter of the catheter change to $Do=3.2\sim3.3$ mm. Finally, a rubber tube with the outer/inner diameter of 3.0/2.5 mm is glued with the proximal end of the printed catheter using the silicone glue. The assembled prototype is shown in **Figure S**1b.

**Supporting Information**

Supporting Information is available from the Wiley Online Library or from the author.

Acknowledgments

This work was support by the National Science Foundation of China (61922093, U1813211, and 61773326) and Hong Kong RGC General Research Fund (CityU11214817) and Sichuan provincial-Hong Kong university cooperation project 2019YFSY0007.

Received: ((will be filled in by the editorial staff))
Revised: ((will be filled in by the editorial staff))
Published online: ((will be filled in by the editorial staff))

References

[1] M. Cianchetti, C. Laschi, A. Menciassi, P. Dario, *Nat. Rev. Mater.* **2018**, *3*, 143.

[2] G.-Z. Yang, J. Bellingham, P. E. Dupont, P. Fischer, L. Floridi, R. Full, N. Jacobstein, V. Kumar, M. McNutt, R. Merrifield, *Sci. Rob.* **2018**, *3*, eaar7650.

[3] M. Runciman, A. Darzi, G. P. Mylonas, *Soft Rob.* **2019**, *6*, 423.

[4] T. Kato, I. Okumura, H. Kose, K. Takagi, N. Hata, *Int. J. Comput. Assist. Radiol. Surg.* **2016**, *11*, 589.

[5] W. Xu, J. Chen, H. Y. K. Lau, H. Ren, *Int. J. Med. Robot.* **2017**, *13*, e1774.

[6] W. Xu, C. C. Poon, Y. Yam, P. W. Chiu, *Int. J. Med. Robot.* **2017**, *13*, e1747.

[7] K. H. Lee, D. K. C. Fu, M. C. W. Leong, M. Chow, H. C. Fu, K. Althoefer, K. Y. Sze, C. K. Yeung, K. W. Kwok, *Soft Robot*. **2017**, *4*, 324.

[8] A. Arezzo, Y. Mintz, M. E. Allaix, S. Arolfo, M. Bonino, G. Gerboni, M. Brancadoro, M. Cianchetti, A. Menciassi, H. Wurdemann, *Surgical endoscopy*. **2017**, *31*, 264.

[9] B. Mosadegh, P. Polygerinos, C. Keplinger, S. Wennstedt, R. F. Shepherd, U. Gupta, J. Shim, K. Bertoldi, C. J. Walsh, G. M. Whitesides, *Adv. Funct. Mater.* **2014**, *24*, 2163.

[10] P. Polygerinos, N. Correll, S. A. Morin, B. Mosadegh, C. D. Onal, K. Petersen, M. Cianchetti, M. T. Tolley, R. F. Shepherd, *Adv. Eng. Mater.* **2017**, *19*, 1700016.

[11] H. Lu, Y. Hong, Y. Yang, Z. Yang, Y. Shen, *Adv. Sci.* **2020**, *7*, 2000069.

[12] H. Lu, M. Zhang, Y. Yang, Q. Huang, T. Fukuda, Z. Wang, Y. Shen, *Nat Commun*. **2018**, *9*, 3944.

[13] J. Edelmann, A. J. Petruska, B. J. Nelson, *The International Journal of Robotics Research*. **2017**, *36*, 68.

[14] J. Edelmann, A. J. Petruska, B. J. Nelson, *Journal of Medical Robotics Research*. **2018**, *03*, 1850002.

[15] S. Jeon, A. K. Hoshiar, K. Kim, S. Lee, E. Kim, S. Lee, J. Y. Kim, B. J. Nelson, H. J. Cha, B. J. Yi, H. Choi, *Soft Robot*. **2019**, *6*, 54.

[16] Y. Kim, A. P. German, S. Liu, X. Zhao, *Sci. Rob.* **2019**, *33*, eaax7329.

[17] J. Z. Gul, Y. J. Yang, K. Y. Su, K. H. Choi, *Soft Robot*. **2017**, *4*, 224.

[18] M. Sivaperuman Kalairaj, B. S. Yeow, C. M. Lim, H. Ren, *Med. Biol. Eng. Comput.* **2020**, *58*, 611.

[19] R. Kang, D. T. Branson, T. Zheng, E. Guglielmino, D. G. Caldwell, *Bioinspiration Biomimetics*. **2013**, *8*, 036008.

[20] A. Shiva, A. Stilli, Y. Noh, A. Faragasso, I. D. Falco, G. Gerboni, M. Cianchetti, A. Menciassi, K. Althoefer, H. A. Wurdemann, *IEEE Rob. Autom. Lett.* **2016**, *1*, 632.

[21] J. Liu, J. Wei, G. Zhang, S. Wang, S. Zuo, *J. Mech. Des.* **2019**, *141*, 082302.

[22] X. Hu, A. Chen, Y. Luo, C. Zhang, E. Zhang, *Comput Assist Surg* **2018**, *23*, 21.

[23] R. Zhao, Y. Kim, S. A. Chester, P. Sharma, X. Zhao, *J. Mech. Phys. Solids*. **2019**, *124*, 244.

[24] Y. Kim, H. Yuk, R. Zhao, S. A. Chester, X. Zhao, *Nature*. **2018**, *558*, 274.

**
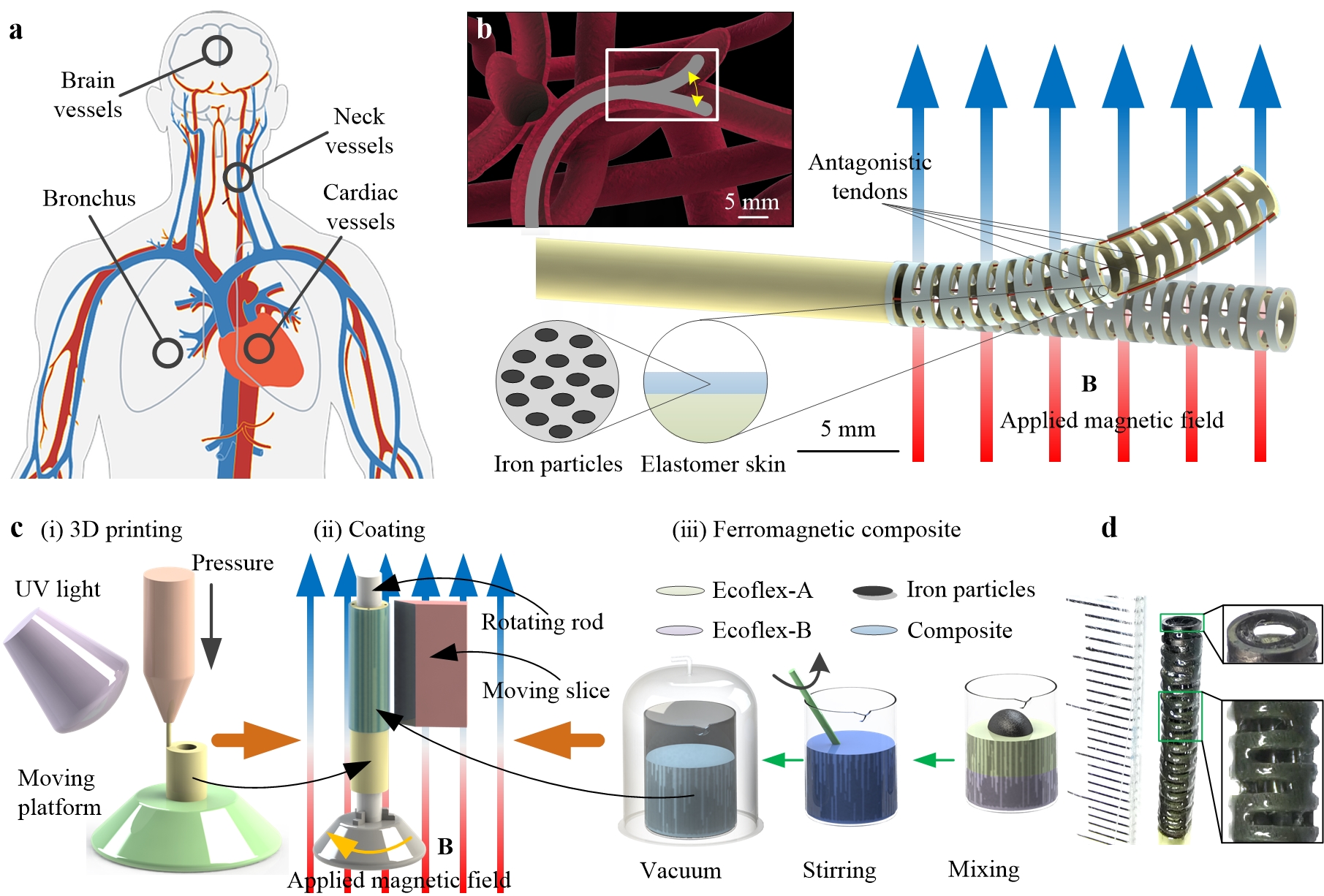
**

**Figure 1.** Illustration of small-scale soft continuum robot design with hybrid magnetic & tendon driven mode. (a) Pathologic areas across the human body hard-to-reach, where small-scale soft steering robots can present their superiority. (b) Illustration of steering ability of a small-scale soft continuum robot catheter passing through a complex constrained vascular environment. The enlarged schematic of the magnetic & tendon driven soft continuum robot with an elastomer skin grown onto the hollow skeleton. The elastomer skin consists of silicone rubber embedded with evenly rearranged iron particles. (c) The schematic illustration of the fabrication process for the soft continuum robot. (ⅰ) The micro 3D printing process by which the detailed structure of the catheter skeleton is fabricated. (ⅱ) The illustration of the coating process. The ferromagnetic elastomer skin is brushed onto the outer surface of the continuum robot and rearranged under external applied magnetic field **B**. (ⅲ) The procedures of making ferromagnetic elastomer skin which consists of silicone rubber (Ecoflex) and micro iron particles with a prescribed mass fraction. (d) The optical image of the fabricated soft continuum robot. The spacing here represents one millimeter.


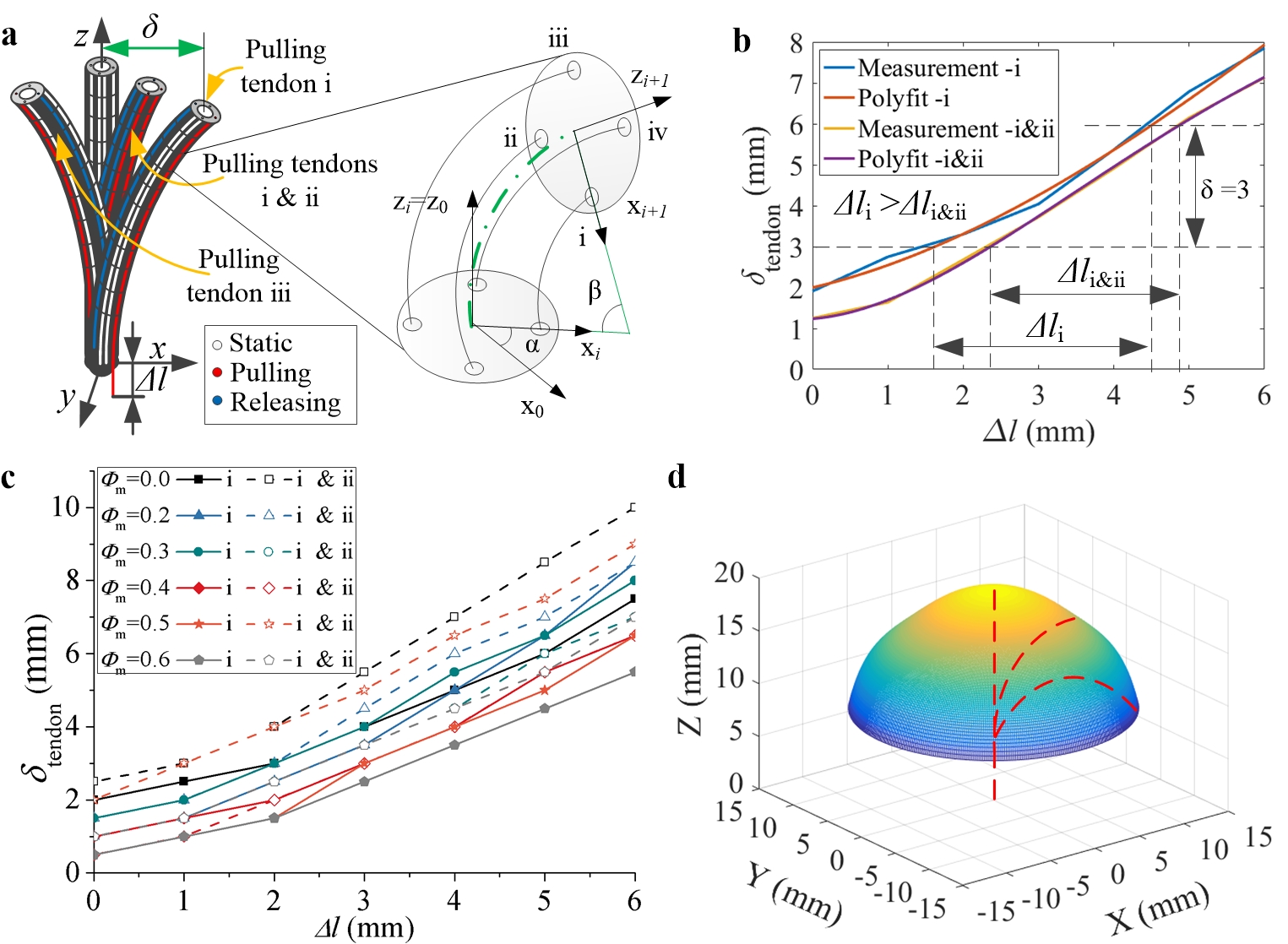


**Figure 2.** Properties of soft continuum robot under the tendon-driven mechanism. (a) Sketch of the different postures of robots against varied tendon-driven strategies. The enlarged part represents the theoretical model of continuous single-joint of the catheter, established by using the piecewise constant curvature approximation (PCCA) model. The characters ⅰ, ⅱ, ⅲ, and ⅳ represent the corresponding tendon. (b) Deflection of catheter-tip against tendon's elongation obtained from both experimental measurement and analytic curve-fitting when the robot is actuated by single and adjacent pairs of tendons. The characters ⅰ, and ⅰ&ⅱ denote the actuation strategy of single-pair and adjacent-pair tendons, respectively. (c) The experimental measurement of the catheter-tip's deflection against tendon's elongation under different pull-release strategies for the varied mass fraction of iron particles. (d) Prediction of workspace, obtained by utilizing the structural symmetry, of catheter-tip of the soft continuum robot.


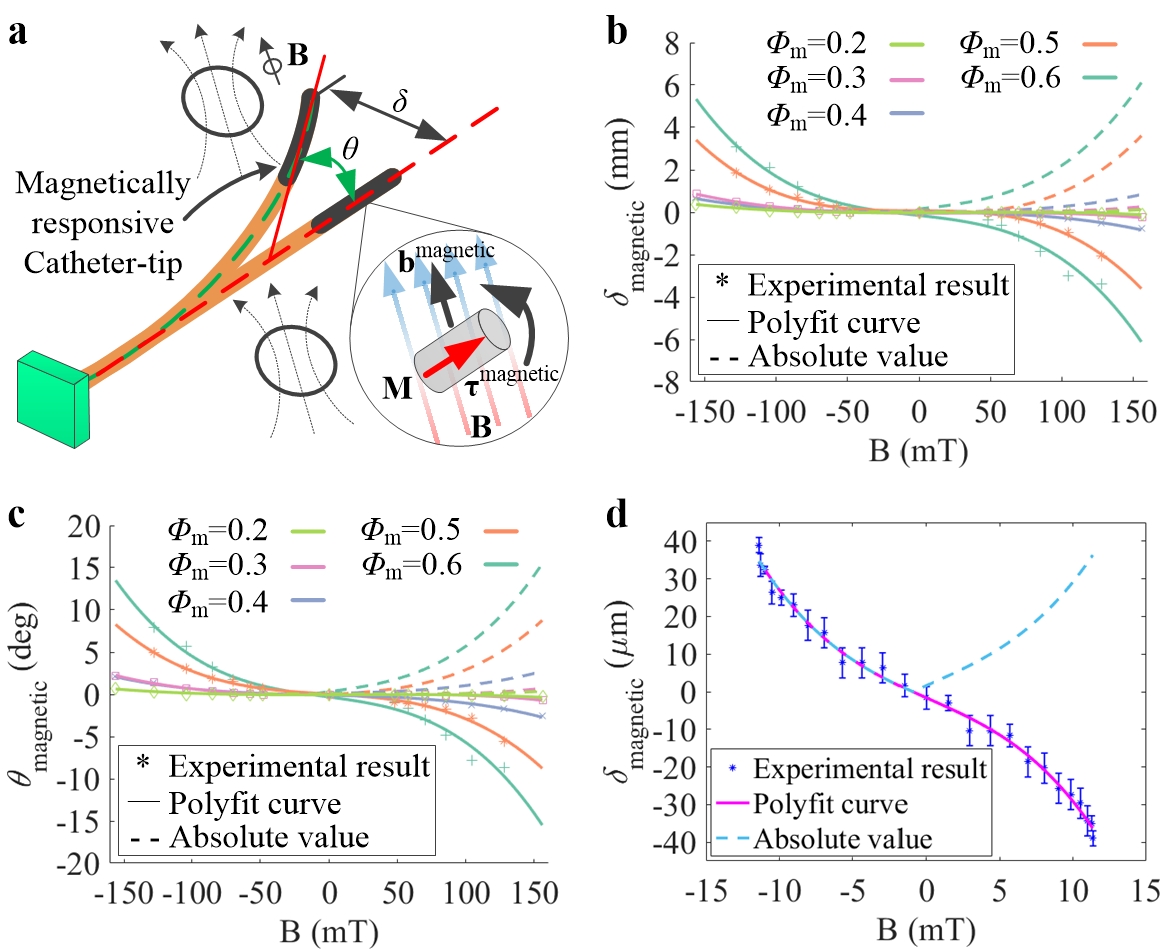


**Figure 3.** Optimal design of magnetic positioning for the soft continuum robot. (a) Sketch of the proposed soft continuum robot under the actuation of an external applied magnetic field. (b) Experimental measurement and the corresponding curve fitting of the deflection against the magnitude of the applied magnetic field, the catheter-tip grow elastomer skin embedded with a different mass fraction of iron particles. (c) Experimental measurement and the corresponding curve fitting for the actuation angle against the magnitude of the applied magnetic field, the catheter-tip grown elastomer skin embedded with different iron particles mass fraction. (d) Experimental measurement and the corresponding curve fitting for the deflection of catheter-tip actuated by a controllable magnetic field. The robot tested in this experiment owns the mass fraction of 0.6 for iron particles.


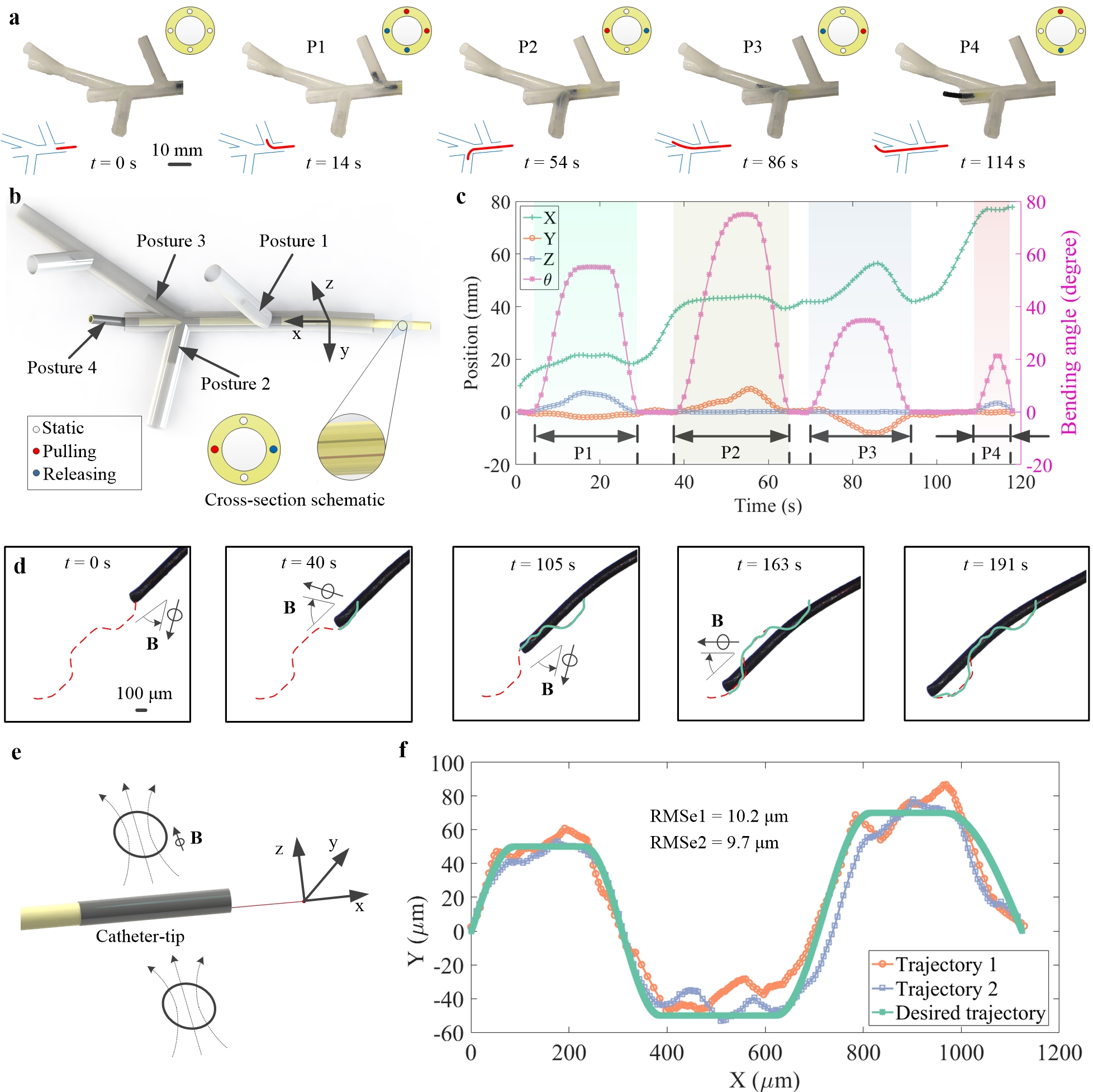


**Figure 4.** Demonstration of active steering and positioning capabilities of soft continuum robot. (a) Experimental demonstration of selectively steering within a tunnel with a series of bifurcation sections, when the robot under the tendon-driven mechanism. (b) Schematic demonstration of different postures of the proposed soft continuum robot when passing through a branched tunnel with an active catheter-tip actuated by tendons. (c) Experimental measurement of the position and bending angle of the catheter-tip under tendon-driven mechanism during the entire passing period. (d) Experimental process of high-precision positioning of the robot under actuation of the external applied magnetic field. (e) Schematic demonstration of the robot positioning under the external applied controllable magnetic field. (f) Experimental measurement of the trajectories of the catheter-tip under magnetic actuation. The robot shows the high-precision navigation capacity, with an external magnetic field applied at the distal magnetically responsive tip. The proximal end of the robot was pushed to advance the entire body during the whole steering and navigating process. Detailed dimensions of the demonstration setup are available in the supplemental material (**Figure S4**a).


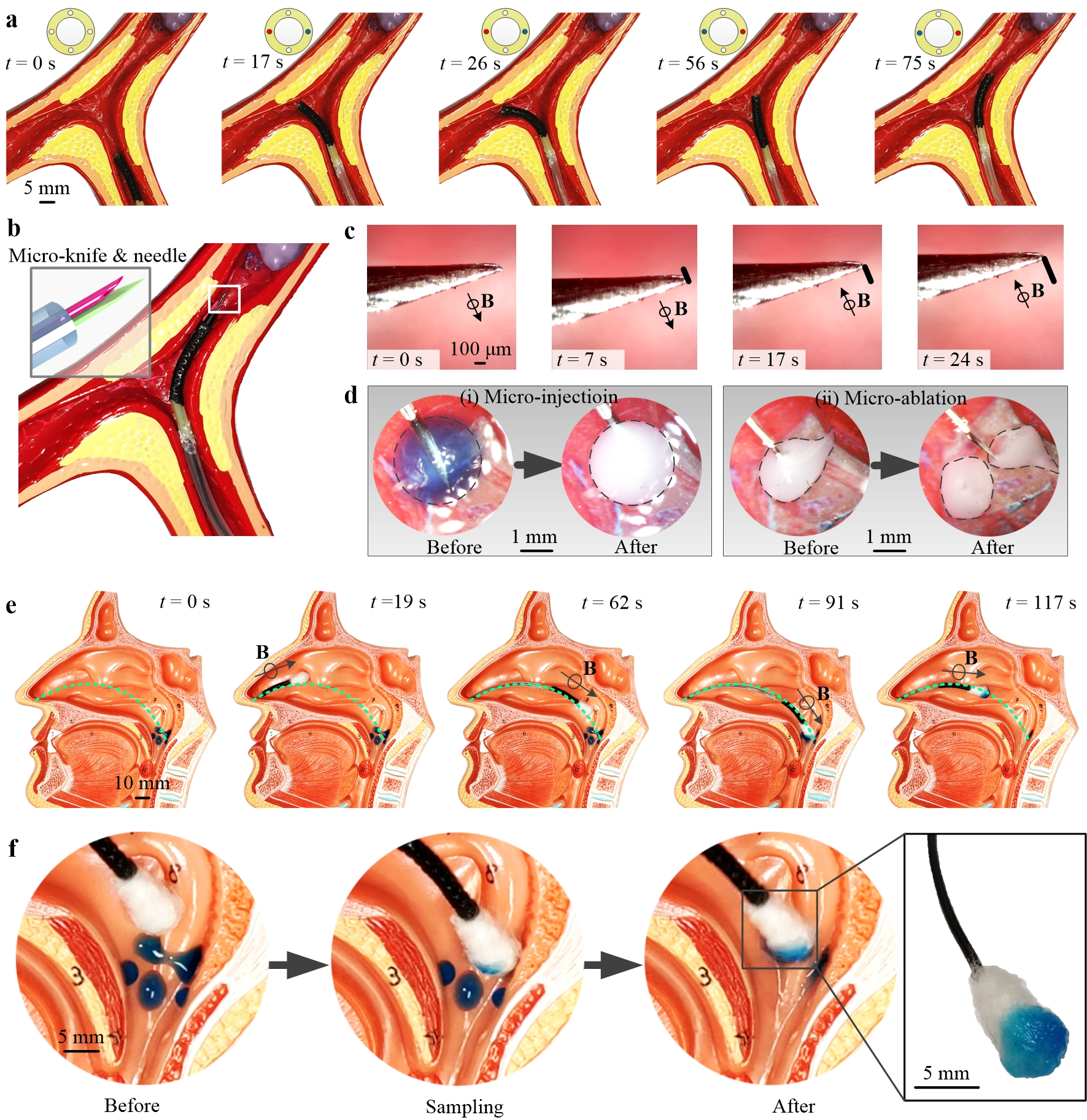


**Figure 5.** Demonstration of manipulation within the vessel model and the process of nasopharyngeal sampling. (a) Demonstration process of active steering within a vessel model of the proposed soft continuum robot under the tendon-driven mechanism. (b) Schematic illustration of the soft continuum robot approached pathology area within vessels, with a micro-scale knife and needle incorporated inside the robot. (c) The experimental trajectory of the catheter-tip under magnetic actuation. (d) The potential application of micro-manipulation as target therapy, including (ⅰ) micro-injection and (ⅱ) micro-ablation. (e) Experimental process of the nasopharyngeal sampling within the nasal cavity, with a swab incorporated in the proposed soft continuum robot. (f) The enlarged demonstration of the swab-tip during the sampling process. Detailed dimensions of the demonstration setup are available in the supplemental material (**Figure S4**).

Copyright WILEY-VCH Verlag GmbH & Co. KGaA, 69469 Weinheim, Germany, 2016.

Supporting Information

Millimeter-scale Soft Continuum Robot for Large Angle and High Precision Manipulation by Hybrid Actuation

Tieshan Zhang, Liu Yang, Xiong Yang, Rong Tan, Haojian Lu*, Yajing Shen*

Tieshan Zhang, Liu Yang, Xiong Yang, Rong Tan, Prof. Haojian Lu, Prof. Yajing Shen

Department of Biomedical Engineering

City University of Hong Kong

Tat Chee Avenue, Kowloon, Hong Kong, China
E-mail: haojianlu2-c@my.cityu.edu.hk, [yajishen@cityu.edu.hk](mailto:yajishen@cityu.edu.hk)

Prof. Haojian Lu

The State Key Laboratory of Industrial Control and Technology

Zhejiang University

Hangzhou 310027, China

Email: luhaojian@zju.edu.cn

Prof. Yajing Shen

Shenzhen Research Institute of City University of Hong Kong

Shenzhen 518057, China

E-mail: [yajishen@cityu.edu.hk](mailto:yajishen@cityu.edu.hk)

**This file includes:**

Supplementary Text

Supplementary Figures

Supplementary Tables

Supplementary Videos

**Supplementary Text**

**1. Analytical model for soft continuum robot under a tendon-driven mechanism**

With an actuation strategy of pull-release on each pair of tendons, the robot could achieve the bending degree of freedom along two perpendicular directions. To obtain the relationship between the posture of catheter-tip and the elongation of each tendon, we can use the piecewise constant curvature approximation (PCCA) model to separate the active part of the catheter into multiple sections and establish a geometric model of continuous single-joint (**Figure 2**a).

The base coordinate system of the robot is set as {X*_o_*-Y*_o_*-Z*_o_*}. We construct the coordinate system {O*_i_*} and {O*_i+1_*} in the center of the upper plane of the *i*th and *i+1*th section, respectively. The axes of Z are perpendicular to the upper plane of each section, while the axes of X point to the hole of the first drive tendon. Besides, the Y-axis of each coordinate system is determined by the right-hand rule. Given the condition that the X*_i_* poses a rotation angle, i.e. *α*, with the X*_o_* along Z*_i_* and that Z*_i_* keeps the same direction of Z*_o_*. Then, we can get the expression of transformation matrix *T* from O*_i_* to O*_i+1_* as following:

$\begin{matrix} T=\text{Trans(}\frac{l}{\beta}\text{cos}\alpha\text{(1-cos}\beta\text{),}\frac{l}{\beta}\text{sin}\alpha\text{(1-cos}\beta\text{),}\frac{l}{\beta}\text{sin}\beta\text{)Rot(z,}\alpha\text{)Rot(y,}\beta\text{)Rot(z,-}\alpha\text{)} \\ \text{=}\left[\begin{matrix} c^{2}\alpha c\beta+s^{2}\alpha& s\alpha c\alpha c\beta-s\alpha c\alpha& c\alpha s\beta& \frac{l}{\beta}c\alpha(1-c\beta) \\ s\alpha c\alpha c\beta-s\alpha c\alpha& s^{2}\alpha c\beta+c^{2}\alpha& s\alpha s\beta& \frac{l}{\beta}s\alpha(1-c\beta) \\ -c\alpha s\beta& -s\alpha s\beta& c\beta& \frac{l}{\beta}s\beta\\ 0 & 0 & 0 & 1 \end{matrix}\right] \end{matrix}$ (S1)

where *β* represents the bending angle, and *l* denotes the length of the virtual central axis, shown as the green dashed curve in **Figure 2**a. For simplifying the form of the transformation matrix, we can express it as below:

$T\text{=}\left[\begin{matrix} n_{x} & o_{x} & a_{x} & p_{x} \\ n_{y} & o_{y} & a_{y} & p_{y} \\ n_{z} & o_{z} & a_{z} & p_{z} \\ 0 & 0 & 0 & 1 \end{matrix}\right]$ (S2)

where ***n, o, a*** represent the unit vector of the *i*th coordinate system, respectively, while ***p*** denotes the position vector of the center.

Combining the **Equation S1** and **Equation S2**, we can obtain

$\left\{\begin{matrix} \alpha=\arctan(\frac{p_{y}}{p_{x}}) \\ \beta=\arccos(a_{z}) \end{matrix}\right.$ (S3)

Then, the elongation of each tendon can be expressed as

$\left\{\begin{matrix} \triangle l_{1}=r_{1}\beta\cos\alpha\\ \triangle l_{2}=r_{1}\beta\cos(\alpha-\pi/2) \\ \triangle l_{3}=r_{1}\beta\cos(\alpha-\pi) \\ \triangle l_{4}=r_{1}\beta\cos(\alpha-3\pi/2) \end{matrix}\right.$ (S4)

where *r*_1_ denotes the distance between the center of the hole (passing through the first tendon) and the center of the plane.

By accumulating the deflection of all actuated sections, we can finally obtain the elongation of tendons for the robot as

$\left\{\begin{matrix} \triangle l_{1}=\sum_{i=1}^{m} r_{1}\beta_{i}\cos\alpha\\ \triangle l_{2}=\sum_{i=1}^{m} r_{1}\beta_{i}\cos(\alpha-\pi/2) \\ \triangle l_{3}=\sum_{i=1}^{m} r_{1}\beta_{i}\cos(\alpha-\pi) \\ \triangle l_{4}=\sum_{i=1}^{m} r_{1}\beta_{i}\cos(\alpha-3\pi/2) \end{matrix}\right.$ (S5)

where *m* denotes the number of all deflected sections of the robot, and *β_i_* represents the bending angle of each section.

As for the PCCA model, the bending angles of all sections have been viewed as equal. Then, the theoretical expressions of elongation of each tendon for the proposed robot with 10 sections can be described as

$\left\{\begin{matrix} \triangle l_{1}=10r_{1}\beta\cos\alpha\\ \triangle l_{2}=10r_{1}\beta\cos(\alpha-\pi/2) \\ \triangle l_{3}=10r_{1}\beta\cos(\alpha-\pi) \\ \triangle l_{4}=10r_{1}\beta\cos(\alpha-3\pi/2) \end{matrix}\right.$ (S6)

**2. Analytical model for soft continuum robots actuated by a magnetic field**

As depicted in **Figure 3**a, the volume unit within the elastomer skin will bear both magnetic body torque $\mathbf{\tau}^{\text{magnetic}}$ and magnetic body force $\mathbf{b}^{\text{magnetic}}$, when the robot is placed under the external magnetic field. The deformation of the catheter-tip results from the coupling effect of magnetic torque and force. To simplify the mechanical status of the robot, a model ^[14]^ based on the magnetic Cauchy stress would be applied to carry out the theoretical analysis. The applied model would cause the same deformation as that of magnetic body torque and force. We can denote the induced magnetization at any point of this continuum robot in an undeformed configuration as a vector **M**, along the axial direction. The deformation gradient, deformed by applied external magnetic field **B**, at the corresponding point could be represented as **F**. Take the theoretical framework of ferromagnetic soft materials ^[23,24]^ as references, the deformation could be calculated by the magnetic Cauchy stress according to the following equation

$\mathbf{\sigma}^{magnetic}=-\mathbf{B}\otimes\mathbf{FM}$ (S7)

where represents the dyadic product.

As for the small volume and thin-wall structure of the robot, the gravitational body force could be neglected when compared with the magnetic force under an external magnetic field. Assuming the active catheter conforms to the case of small deflection, meaning the free end generates a bending smaller than 10% of the length. When the catheter is placed under a magnetic field **B** perpendicular with **M** (**Figure 1**c), there is only shear stress of the entire magnetic Cauchy stress tensor left. ^[23]^ The shear stress will contribute to the deformation of the catheter. For the undeformed status of the catheter (i.e. **F** = **1**), the shear stress can be expressed as $\sigma^{magnetic}=-MB$, which further leads to the magnetically induced moment $dM=-MBdV$, where $dV$ denotes an incremental volume of the catheter. To obtain the maximal deflection, the effective moment could also be viewed as a result caused by a point force *F* applied at the free end of the robot. With a direction same as the external magnetic field, the force can be analytically presented as:

$F=\left|\frac{dM}{dx}\right|=\left|\frac{-MBdV}{dx}\right|=\left|\frac{-MBAodx}{dx}\right|=MBAo$ (S8)

where $Ao=\pi(Do^{2}-D^{2})/4$ denotes the cross-sectional area of the ferromagnetic elastomer skin, and *dx* defines the incremental length along the robot.

To facilitate subsequent calculations, we simplify the robot as a uniform tube with the following geometry dimensions, including length *L*, outer diameter *Do*, and inner diameter *d*. Thus, the cross-sectional area and moment of inertia can be expressed as $A=\pi(Do^{2}-d^{2})/4$ and $I=\pi(Do^{4}-d^{4})/64$, respectively. The bending stiffness of the simplified catheter can be stated as $K=3EI/L^{3}$, where *E* denotes Young’s modulus of the composite material. Also, there is a simplification for an incompressible object undergoing small deflection that $E=3G$, where *G* defines the shear modulus of the material. We can finally reach the analytical expression for the deformation of the distal tip as following

$\delta_{\text{magnetic}}=\frac{F}{K}=\frac{16L^{3}(Do^{2}-D^{2})}{9(Do^{4}-d^{4})}\frac{MB}{G}$ (S9)

**3. Analytical model for optimizing mass fraction of iron particles**

As both the magnitude of induced magnetization and shear modulus of the catheter would be affected by the mass fraction, denoted by *ϕ_m_*, of iron particles. Given a certain magnitude of external applied magnetic field *B*, the deflection of the free end would only vary with the mass fraction.

With the pulling force applied at the proximal end of tendons, the catheter-tip would generate bending. The radial component of pulling force applied at the distal tip of the robot through tendon could be simplified as a point force perpendicular with the longitudinal axis, and the deflection can be further approximately equivalent with the bending of a cantilever beam under a concentrated force. The maximum deflection at the tip can be expressed as

$\delta_{\text{tendon}}=\frac{1}{144}\frac{F_{\text{eq}}}{G}\frac{L^{3}}{I}$ (S10)

where *F*_eq_ denotes the simplified point force. According to **Equation** **S**10, the expression of shear modulus can be obtained

$G=\frac{L^{3}}{144I}\frac{F_{\text{eq}}}{\delta_{\text{tendon}}}$ (S11)

According to **Figure 2**c, it is obvious that more elongation would be needed to reach the same deflection as particle mass fraction increasing. There should be a proportional relationship between the point force and the elongation of tendons, i.e. $F_{\text{eq}}\sim\triangle l$. Thus, assuming that all catheters own the same geometric dimensions, for achieving the same deflection, more elongation means the up-rising trend of the shear modulus of the robot body as the particle mass fraction increasing, i.e. .

According to **Equation** **S**9, we know that the deflection caused by an external magnetic field is mainly affected by both the external magnetic magnitude and its material, i.e. $\delta_{\text{magnetic}}\text{=}C_{1}(\frac{M}{G}B)$. According to **Figure 3**b, the robot embedded with a higher mass fraction of iron particles generates a bigger deflection under the same magnitude of the magnetic field. There is a sharp increase of deflection when the mass fraction is larger than 40%, which means the material-related variable *M* /*G* presents the same changing trend with particle mass fraction, i.e. . Taking both shear modulus and the induced magnetization of the robot into consideration, it can be concluded that the increase of induced magnetization *M* of the robot is obviously faster than that of shear modulus *G* as the increasing of mass fraction of iron particles. Finally, the relationship between deflection and particle mass fraction as proportional, i.e., can be obtained. It is concluded that the robot coated with the elastomer skin, which consists of a higher mass fraction of iron particles, will have a more noticeable response under magnetic actuation.

**Supplementary Figures**


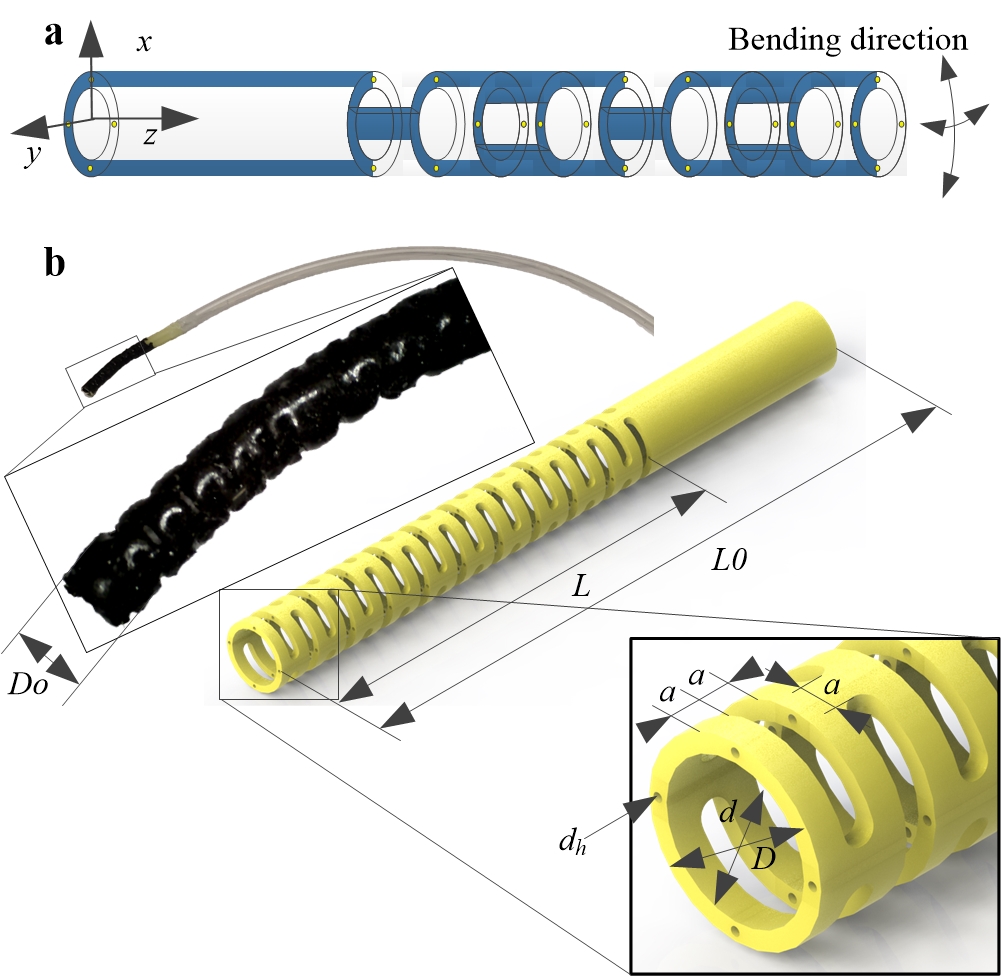


**Figure S1.** A detailed illustration of the structure for the robot. (a) The schematic of the hollow structure which can achieve bending along two vertical directions. (b) The detailed prototype and 3D model of the proposed skeleton of the robot.


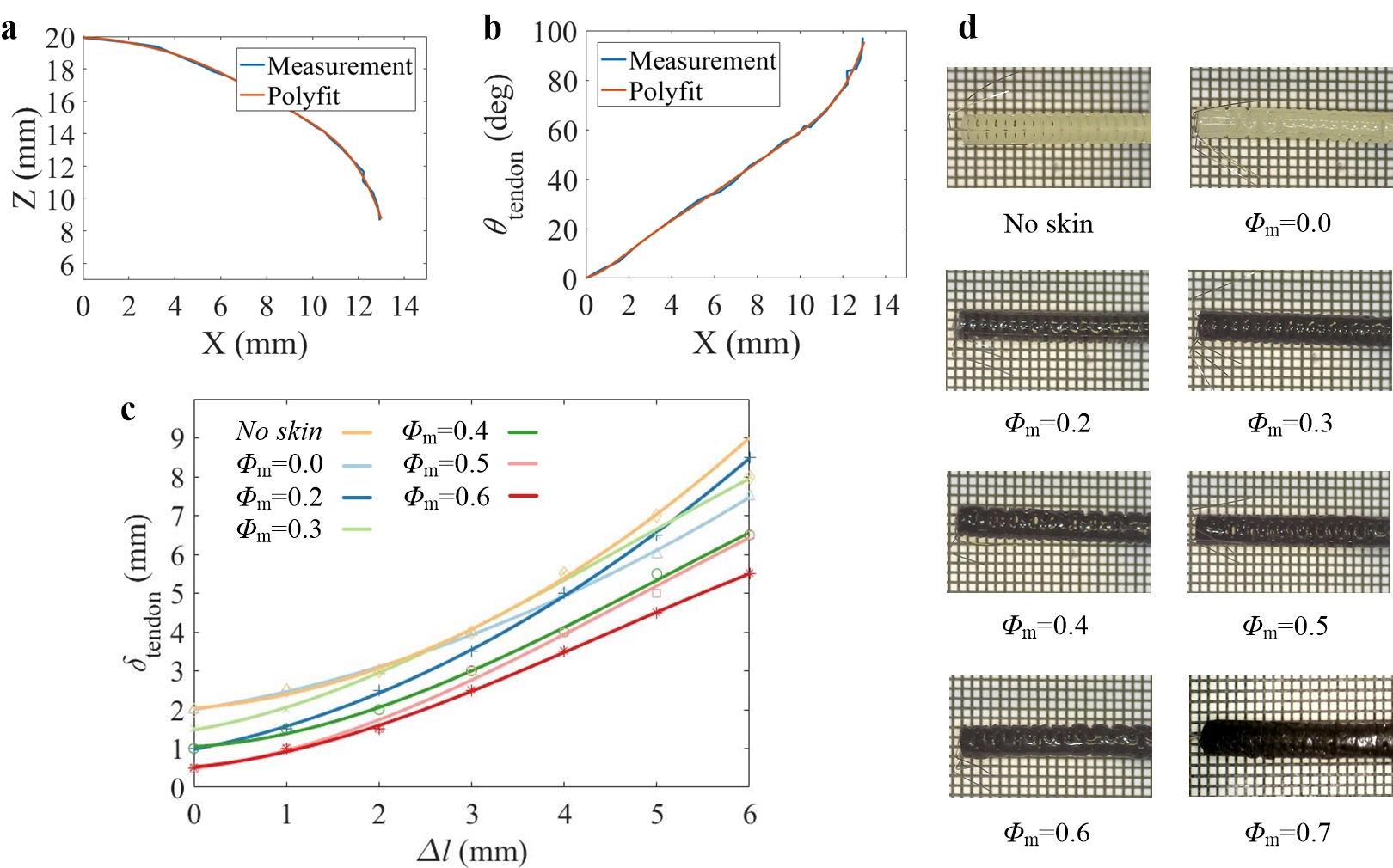


**Figure S2.** The properties of the tendon-driven actuation mechanism. (a) The recorded experimental data and the corresponding analytical curve-fitting of deflection for the catheter-tip. (b) The experimental measurement and analytical curve-fitting of the bending angle for the catheter-tip when actuated by tendons. (c) The experimental measurement and analytical curve-fitting of the catheter-tip's deflection against single-pair tendon's elongation for the varied mass fraction of iron particles. (d) The comparison between different continuum robots with a varied mass fraction of iron particles.


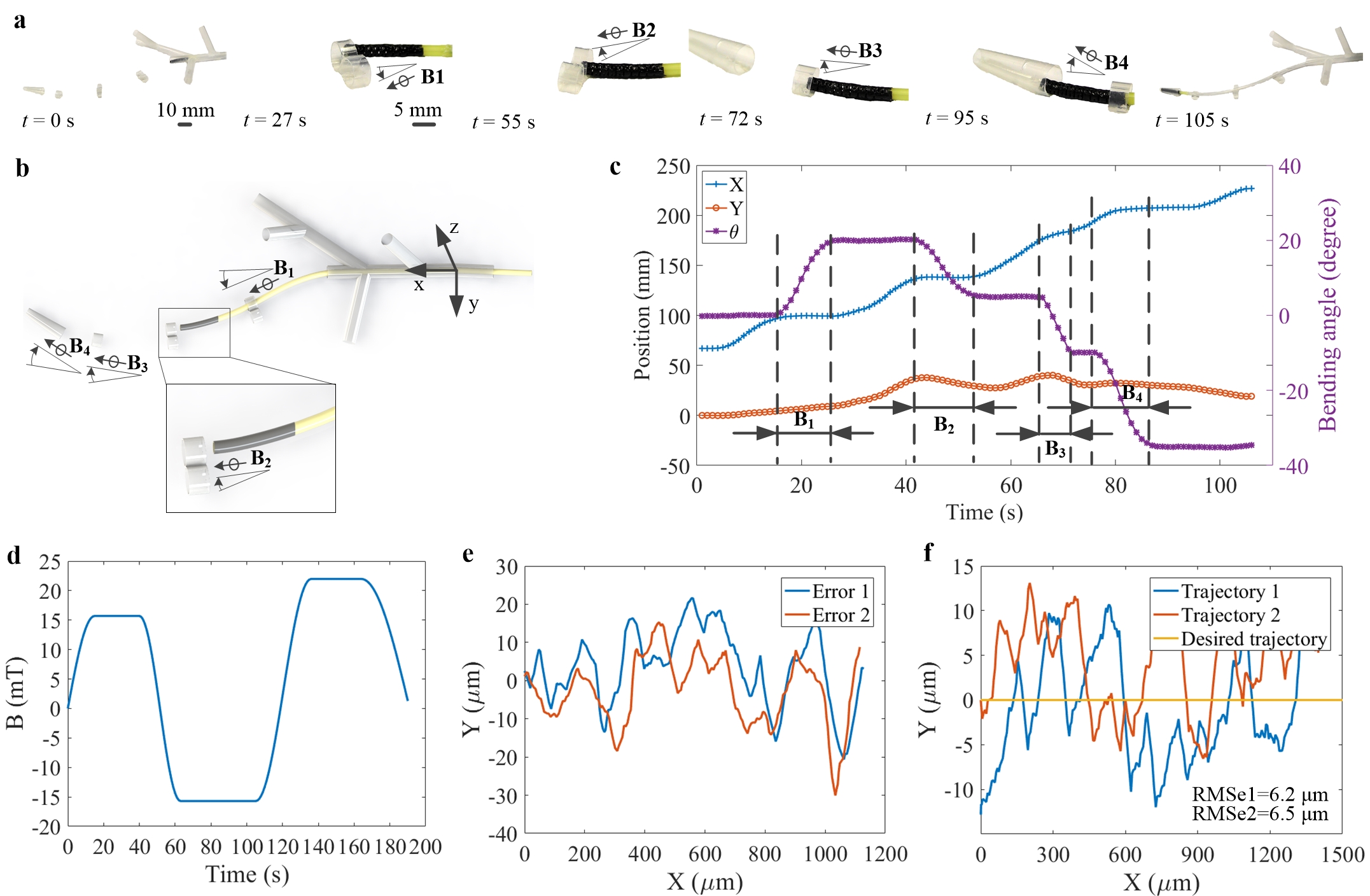


**Figure S3.** Experimental measurement of steering and positioning for the soft continuum robot under magnetic actuation. (a) Experimental steering process of the soft continuum robot under magnetic actuation. (b) Schematic demonstration of the robot steering under the external applied magnetic field. (c) Experimental measurement of the position and bending angle of the catheter-tip under magnetic actuation. (d) The measured relationship between magnetic magnitude and experimental time under the controllable magnetic platform. (e) The error between the actual trajectories and the desired one under the magnetic actuation. (f) The measured linear trajectories of the catheter-tip without being applied magnetic field.


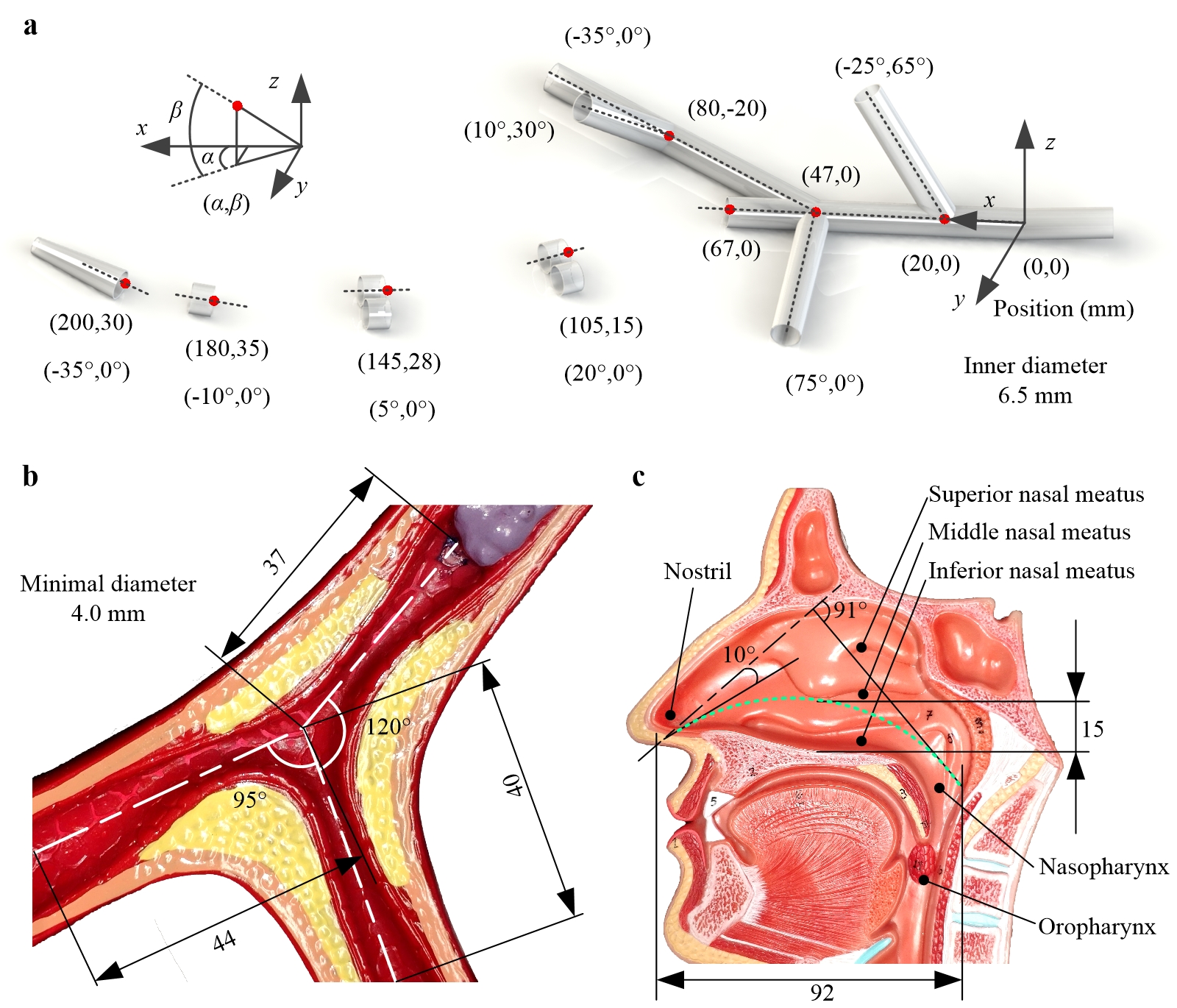


**Figure S4.** Dimensions of the experimental setup. (a) Dimensions of the branched tunnel and series of rings used for the experimental demonstration process of the active steering and navigation functionalities of the proposed soft continuum robot with hybrid-actuation. (b) Dimensions of the vessel model used for the experimental demonstration process of precise manipulation. (c) Dimensions of the nasal cavity model used for the experimental demonstration process of nasopharyngeal sampling.

**Supplementary Tables**

**Table S1.** Mechanical parameters of materials used for the robot

| Material | Elastic module | Tensile strength | Breaking elongation rate | Hardness |
| --- | --- | --- | --- | --- |
| HD | 3.6 GPa | 61.4 MPa | 24.3% | 84 Shore D |
| Ecoflex 20 | 0.33 MPa | 3.79 MPa | 620% | 20 Shore A |

**Table S2.** Geometry parameters of components of the robot

| Object | Catheter | Sheath | Micro-needle | Micro-knife |
| --- | --- | --- | --- | --- |
| Outer diameter  (Hight) | 3.30 mm | 2.00 mm | 0.45 mm | (0.60) mm |
| Inner diameter  (Width) | 2.40 mm | 0.90 mm | 0.30 mm | (0.25) mm |

**Supplementary Videos**

**Video-S1.** Dynamic tracking process under the magnetic actuation

**Video-S2.** Steering process within the vessel model with a tendon-driven mechanism

**Video-S3.** Precise positioning within the vessel model under the magnetic actuation

**Video-S4.** Nasopharyngeal sampling process under the guidance of a magnetic field
